# Estimating the generation interval from the incidence rate, the optimal quarantine duration and the efficiency of fast switching periodic protocols for COVID-19

**DOI:** 10.1038/s41598-022-08197-x

**Published:** 2022-03-17

**Authors:** Eugenio Lippiello, Giuseppe Petrillo, Lucilla de Arcangelis

**Affiliations:** 1 Department of Mathematics and Physics, University of Campania “L. Vanvitelli”, Viale Lincoln, 5, Caserta, 81100 Italy; 2 Department of Engineering, University of Campania “L. Vanvitelli”, Via Roma, 29, Aversa, 81031 Italy

**Keywords:** Complex networks, Nonlinear phenomena

## Abstract

The transmissibility of an infectious disease is usually quantified in terms of the reproduction number $R_t$ representing, at a given time, the average number of secondary cases caused by an infected individual. Recent studies have enlightened the central role played by *w*(*z*), the distribution of generation times *z*, namely the time between successive infections in a transmission chain. In standard approaches this quantity is usually substituted by the distribution of serial intervals, which is obtained by contact tracing after measuring the time between onset of symptoms in successive cases. Unfortunately, this substitution can cause important biases in the estimate of $R_t$. Here we present a novel method which allows us to simultaneously obtain the optimal functional form of *w*(*z*) together with the daily evolution of $R_t$, over the course of an epidemic. The method uses, as unique information, the daily series of incidence rate and thus overcomes biases present in standard approaches. We apply our method to one year of data from COVID-19 officially reported cases in the 21 Italian regions, since the first confirmed case on February 2020. We find that *w*(*z*) has mean value ${\overline{z}} \simeq 6$ days with a standard deviation $\sigma \simeq 1$ day, for all Italian regions, and these values are stable even if one considers only the first 10 days of data recording. This indicates that an estimate of the most relevant transmission parameters can be already available in the early stage of a pandemic. We use this information to obtain the optimal quarantine duration and to demonstrate that, in the case of COVID-19, post-lockdown mitigation policies, such as fast periodic switching and/or alternating quarantine, can be very efficient.

## Introduction

An accurate estimate of transmission parameters is fundamental in monitoring the spreading of a disease during a pandemic^1–9^. The coronavirus disease 2019 (COVID-19) pandemic has shown the relevance of an accurate evaluation of the time-dependent reproduction number $R_t$ to monitor the effect of non-pharmaceutical interventions (NPI) imposed by local governments. $R_t(t)$ represents the average number of secondary cases that each infected individual would infect if the conditions remained as they were at a given time *t*. A decreasing of $R_t$ indicates that the epidemic is in decline with values $R_t(t) < 1$ suggesting that the epidemic may be regarded as being under control at time *t*. In the epidemiological models considered in our study, structured by time since infection, the estimate of $R_t$ depends on the probability distribution *w*(*z*) of the generation time *z*, i.e. the time difference between the dates of infection of successive cases in a transmission chain. Since the times of infection are rarely known, *w*(*z*) is usually approximated by the distribution of the serial intervals, which is the difference in dates of symptom onset between a pair of a primary and its secondary case. The serial interval distribution can be obtained via contact tracing and data collected for COVID-19, in different geographic areas, indicate that its mean value ranges from 4 to 7.5 days, with a standard deviation $\sigma _s$ ranging between 3 days up to 6 days inside each area^10–12^. Recent studies^13–15^, however, suggest that, whilst the average value of generation times ${\overline{z}}$ is well approximated by the mean value of serial intervals, the standard deviation $\sigma$ of generation times is significantly smaller than $\sigma _s$. This difference hinders the possibility to correctly tune the optimal duration of quarantine $t_Q$ and also to plan optimized lock-down strategies. For example, $t_Q$ should be reasonably larger than ${\overline{z}}+\sigma$, in order to significantly quench existing infections. Furthermore, it has been recently shown^16,17^ that the distribution of serial intervals obtained from contact tracing is altered by NPI and, in particular, the mean serial interval becomes smaller when NPI are enhanced. This introduces a bias in the estimate of $R_t$, which could be avoided if the parameters controlling the functional form of *w*(*z*) were directly extrapolated from the infection data. An attempt in this direction can be found in Wallinga and Teunis (WT)^18^. In their seminal paper WT propose a method based on the maximization of the likelihood *L*, formulated in terms of *w*(*z*) and of the infection network, in which the nodes represent cases and the directed edges between the nodes represent transmission of infection between cases. White and Pagano (WP)^19^ have subsequently proposed a simpler formulation for *L*, expressed only in terms of the daily series of incidence rate *I*(*t*), i.e. the rate of infected individuals at the calendar time *t*. Their study, however, is restricted to the evaluation of *w*(*z*) in the case of a constant reproduction number $R_t(t)=R_0=const$.

Here we present a generalization of the WP approach which allows us to extract from *I*(*t*) the temporal evolution of $R_t(t)$ together with the optimal functional form of *w*(*z*) consistent with the epidemic curve. More precisely, we are able to explore the whole range of parameters controlling *w*(*z*) and to identify the optimal ones. Our procedure also provides as an output the daily number of imported infected cases $\mu (t)$, which can be implemented in numerical simulations to test the efficiency of the procedure. We discuss in details the results of our approach using data for the COVID-19 disease in Italian regions, taking also into account the influence of periodicity in the collection of epidemic data. Finally, we enlighten the relevance of the information contained in *w*(*z*) in order to design alternative strategies for virus containment in absence of vaccines.

## Basic definitions

A key quantity in outbreak analyses is the transmissibility $$\beta (t,s)$$, defined in such a way that $$\beta (t,s)dt$$ is the average number of people someone, infected at the calendar time *s*, infects during the subsequent time interval $$[t,t+dt)$$, where *dt* is a small time interval. In the following we always assume that the time *t* is larger or equal to the time *s*. Under stationary conditions $$\beta (t,s)$$ is time translationally invariant and it is usually written as $$\beta (t,s)=R_0 w(t-s)$$, where $$R_0$$ is the reproduction number, defined as the average number of secondary cases per primary case over a fully susceptible population. The quantity $$w(z=t-s)$$ is the generation time distribution ($$\int _0^{\infty } dz w(z)=1$$), i.e. the distribution of how infection events are distributed as a function of time since the infection. Nevertheless, in the large majority of cases, the hypothesis of stationarity does not hold, mostly because of changes in contact patterns also induced by public health measures such as social distancing or case isolation. Furthermore also the change in the percentage of susceptible individuals, including the one produced by vaccinations, is responsible for the dependence of $$\beta (t,s)$$ on both times *t* and *s*. For this reason one usually introduces an effective reproduction number $$R_t(t)$$ by assuming $$\beta (t,s)=R_t(t) w(t-s)$$, where $$w(t-s)$$ still represents the normalized generation time distribution “in an ideal large closed setting where contact rates are constant”^20^. The basic idea below this approximation is that $$w(t-s)$$ is an *intrinsic* distribution^17^ which reflects the biological process of disease progression and whose functional form remains substantially unaltered over time. All the changes in the contact structure of the population are conversely described by the temporal evolution of $$R_t(t)$$ which, for instance, provides information about the efficiency of NFI. In a different, but substantially equivalent formulation, one introduces the case reproduction number $$R_c(s)$$, defined by $$\beta (t,s)=R_c(s) w(t-s)$$, representing the average number of people someone infected at time *s* can expect to infect at subsequent times. The two quantities $$R_t(t)$$ and $$R_c(t)$$ are intimately connected

1 $$\begin{aligned} R_c(s)=\int _{s}^{\infty } dt R_t(t)w(t-s) \end{aligned}$$

which indicates that $R_c(s)$ is a smoothed function of $R_t(t)$. The standard procedure adopted to evaluate $R_c(s)$ is based on the algorithm developed by Wallinga-Teunis (WT) [see Eq. (5) in “Methods”]^18^ whereas the direct evaluation of $R_t(t)$ can be obtained by means of the algorithm developed by Cori et al.^21^. These two algorithms have been recently combined to improve the estimate of the effective reproduction number during temporal periods of small incidence^22^.

**Figure 1:**
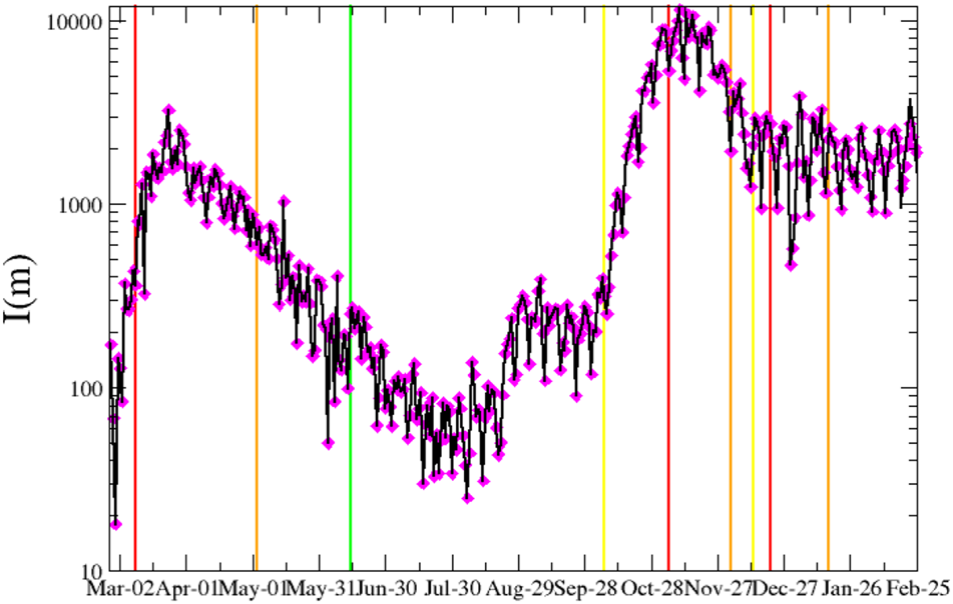
Black continuous lines represent the daily incidence of COVID-19 for the Lombardy from 02/24/2020 up to 02/24/2021. Color vertical lines indicate the starting time of different containment measures, which combines lockdown restrictions and closures with measures such as testing policy and contact tracing, etc. We adopt a color code ranging from red, orange, yellow up to green as a rough indicator of the severity of these restrictions, decreasing from red to green, i.e strong restrictions are imposed in the temporal period after a red line whereas weak ones after a green one. Magenta diamonds represent the result of numerical simulations implementing the best estimate for $\{R_c\}$ and $\{\mu \}$, for $\tau =0.15$, $a=6.2\tau$, provided by the *LL* maximization procedure. The overlap with experimental data is achieved setting $I(0)=250$.

All the above mentioned algorithms assume that *w*(*z*) is already known. This represents a big problem in the early stage of a disease when specific data for *w*(*z*) are not available. Early studies of COVID-19, for example, implement the functional form of *w*(*z*) obtained in previous similar diseases such as SARS or MERS^23,24^, which however presented a mean generation time much larger than the one subsequently found for COVID-19. As soon as specific data become available, the standard approach is represented by the identification, via contact tracing, of the correct infector-infectee pairs. Unfortunately, the accurate timing when an individual is infected is very difficult to be established and generation intervals are often replaced by serial intervals $$s_{ij}$$, defined as the interval between times of onset of symptoms in the infector *i* and in the infectee *j*. More precisely, indicating with $$z_{ij}$$ the generation time between an infector *i* and an infectee *j* and introducing $$q_i$$ as the incubation period of the infector *i* ($$q_j$$ is the incubation period of *j*), one has^15,25^ $$ s_{ij}=z_{ij}+q_j-q_i$$ (see Fig. 1 in^15^ for an illustration). Under the assumption that $$q_i$$ and $$q_j$$ are independent and identically distributed variables, the approximation of *w*(*z*) with the serial interval distribution is reasonable only if one is interested in the mean values $${\overline{z}}$$ of $$z_{ij}$$, which is expected to roughly coincide with the mean value of $$s_{ij}$$. On the other hand, because of the large variability of incubation times, the standard deviation of $$s_{ij}$$ ($$\sigma _s$$) is expected to be significantly larger than the standard deviation of $$z_{ij}$$ ($$\sigma $$) and the serial interval distribution is expected to be much broader than *w*(*z*). As an example, under the assumption that $$z_{ij}$$ is uncorrelated to both $$q_i$$ and $$q_j$$ one obtains $$\sigma _s=\sqrt{2\sigma _q^2+\sigma }$$ where $$\sigma _q$$ is the standard deviation of $$q_i$$. It has been recently shown that approximating *w*(*z*) with the serial interval distribution can lead to a systematic underestimate of $$R_t(t)$$^26^. In order to overcome this problem, in ref.^13–15^ elegant methods based on log-likelihood maximization have been developed to infer the generation time from intervals of exposure and onset of symptoms. These approaches lead to an estimate of $$\sigma $$ which is much smaller than the value of $$\sigma _s$$, previously obtained from the serial interval distribution of the corresponding data set. More precisely, these methods give^13^ for Singapore $$\sigma =1.72$$ to be compared to $$\sigma _s=4.32$$ and for Tianjin $$\sigma =1.51$$ to be compared to $$\sigma _s=4.24$$. At the same time, combining 5 different data-sets it has been obtained^15^ $$\sigma =1.8$$ to be compared with $$\sigma _s=3.8$$. These results, however, explicitly depend on the functional form of the distribution of $$q_i$$ and it is not possible to exclude that they can have been affected by the erroneous identification of the correct infector-infectee pair. This becomes more probable when the percentage of asymptomatic individuals becomes higher and one is often compelled to consider small statistical samples containing a limited number of pairs, whose infection history is well under control. For example, in the case of Lombardy, considered in our study, the previous estimate of $$\sigma _s$$ (Ref.^11,12^) was achieved with a sample containing 90 observations of individual serial intervals divided in 55 clusters. As further examples ref.^27^ considers 66 infector-infectee transmission pairs from China, ref.^28^ considers 18 pairs from Taiwan and ref.^14^ 40 pairs from different geographic areas.

We finally remark that the distribution of time delays between the symptom onset in an infector-infectee pair, measured during the ongoing of an epidemic, can be significantly different from the *intrinsic* serial interval distribution^16,17^. Indeed, it is more probable to observe a shorter time delay when the incidence of primary events is increasing. In the early stage of COVID-19 in China, for example, the mean serial interval decreases from an initial measured value of about 7.8 days to a measured value of about 2.6 days, after one month^16^.

Summarizing, the substitution of *w*(*z*) with the serial interval distribution, measured via contact tracing, is responsible of important biases in the evaluation of $R_t(t)$ during an ongoing epidemic. In the following we present a new method which provides the optimal functional form of *w*(*z*), without resorting to contact tracing. More precisely, the only empirical parameter we consider is the daily incidence which is a discrete series $\{I(m)\}_{m=1,..N}$, where *I*(*m*) represents the daily number of infected individuals recorded on the *m*-th day and *N* is the number of considered days. The key quantity is the daily transmissibility $\beta (m,j)$, representing the average number of infections induced during the *m*-th day by infected cases on the *j*-th day. Accordingly, the daily case reproduction number $R_c(m)$ can be defined as $\beta (m,j)=R_c(j)w(m-j)$, where $w(m)=\int _m^{m+1}w(z)dz$ is the discretized generation time distribution. If the temporal evolution of $\{R_c(m)\}_{m=1, \ldots ,N}$ and the functional form of $w(m-j)$ are assigned, the expected value *E*[*I*(*m*)] of the daily incidence on day *m* can be obtained from the past history according to the renewal equation^1,3,20^

2 $$\begin{aligned} E[I(m)]=\sum _{j=0}^{m-1}R_c(j) w(m-j)I(j) +\mu (m) \end{aligned}$$

where $\mu (m)$ is the daily number of imported cases during the *m*-th day, i.e. infectors coming from outside the considered region. A similar equation can be written in terms of the effective reproduction number $R_t(m)$, however we prefer to consider the case reproduction number $R_c(m)$ which is more suitable for numerical implementation. Indeed, for a given temporal profile of $\{R_c(m)\}_{m=1, \ldots ,N}$ and of $\{\mu (m)\}_{m=1, \ldots ,N}$, we can easily simulate the epidemic curve $\{I(m)\}_{m=1, \ldots ,N}$ from Eq. (2), according to a generation tree algorithm (see “Methods”).

In our procedure, illustrated in the next section, we assume that $w(m-j)$ is stationary in time and we look for the optimal temporal profiles of $\{R_c(m)\}_{m=1, \ldots ,N}$ and of $\{\mu (m)\}_{m=1, \ldots ,N}$ which, implemented in Eq. (2), give an expected value *E*[*I*(*m*)] which is the closest to the observed one *I*(*m*) for each of the *m* days $m \in [1,N]$. After this optimization, achieved by means of a log-likelihood maximization procedure, we identify, among a wide range of possible functional forms of $w(m-j)$, the one which presents the maximum value of the log-likelihood.

## Log-likelihood evaluation

In the generation process the number of individuals infected on the *m*-th day is assumed to be Poisson distributed, $P_P[I(m)]= \frac{E[I(m)]^{I(m)} e^{-E[I(m)]}}{I(m)!}$, with the expected value *E*[*I*(*m*)] obtained from Eq. (2). The likelihood of the time series $\{I(m)\}_{m=1, \ldots ,N}$, for assigned sequences $\{R_c(m)\}_{m=1, \ldots ,N}$, $\{\mu (m)\}_{m=1, \ldots ,N}$ and for a given functional form of $w(m-j)$ is given by $L\left( \{I\},\{R_s\},\{\mu \},\{w\}\right) = \prod _{m=1}^N P_p[I(m)]$. The best series $\{R_c\},\{\mu \},\{w\}$ compatible with the recorded data $\{I\}$ are the ones that maximize the likelihood. We perform this maximization process assuming that the functional form of $w(m-j)$ is assigned and depends on few tuning parameters. More precisely we consider a generation time distribution *w*(*z*) which is either a Gamma, or a Weibull or a log-normal distribution, which are the typical functional forms proposed in the literature^3,10,13–15,29^. For all three distributions, *w*(*z*) is fully characterized by its average value ${{\overline{z}}}$ and the standard deviation $\sigma$, therefore the search for their optimal values is among the main purposes of our approach. This leads to an expression for $L\left( \{I\},\{R_c\},\{\mu \},{{\overline{z}}}, \sigma \right)$ which is equivalent to the one obtained by WP^19^, except that we keep explicitly into account the temporal dependence of $R_c(m)$. Furthermore, we introduce a smoothness constraint on $R_c(m)$, by penalizing its second derivative^30^, in order to impose that $R_c(m)$ does not change abruptly between two subsequent days. The final step in our approach is to consider the logarithm of the likelihood $LL=\log \left( L\right)$ and this allows us to split *LL* into the sum of different terms which can be more easily evaluated, thus providing a more efficient maximization procedure. The final expression for *LL* is given by

3 $$LL\left( \{I(m)\},\{R_c\},\{\mu \},{{\overline{z}}}, \sigma ,V\right) =\sum _{m=1}^N I(m) \log \left( E[I(m)]\right) - \sum _{m=1}^N E[I(m)] -\frac{1}{2 V} \sum _{m=2}^{N-1} \left( R_c(m-1)+R_c(m+1)-2 R_c(m)\right) ^2 $$

where *V* is the parameter that controls the degree of smoothness of $R_c(m)$.

It is worth noticing that, according to Eq. (2), a variation of $R_c(m)$ produces a change of *E*(*I*(*m*)) which is different from the one produced by a variation of $\mu (m)$ and it is therefore possible to discriminate between the role of the two quantities. This idea is at the basis of the method defined stochastic declustering used for instance in seismology to discriminate between triggered and spontaneous earthquakes^31,32^. We have then developed an optimized procedure, based on the Markov-chain-Monte–Carlo method (see “Methods”), which identifies the changes of $\{R_c(m)\}_{m=1, \ldots N}$ and of $\{\mu (m)\}_{m=1, \ldots N}$ that, implemented into Eq. (2), provide values of $\{E(I(m))\}_{m=1, \ldots N}$ which are closer to the experimental $\{I(m)\}_{m=1, \ldots N}$. In particular the method gives, for a fixed *w*(*z*), the best series $\{R_c(m)\}_{m=1, \ldots N}$ and $\{\mu (m)\}_{m=1, \ldots N}$ which correspond to a global maximum of the log-likelihood *LL*^33,34^. Our algorithm is sufficiently fast that we can easily obtain the maximum *LL* for different choices of *w*(*z*) and therefore we are able to explore the full range of relevant values of ${{\overline{z}}}$ and $\sigma$.

In the “Methods” section we also show that our method is not affected by the existence of a temporal delay $\Delta t_{rec}$ between the day of the infection and the day when this infection is identified and recorded in the data set. The presence of this delay, indeed, does not change the renewal equation Eq. (2) under the reasonable assumption that $R_c(m)$ does not change too fast.

Results of our analysis are presented in the next section for the region Lombardy where the first outbreak of Covid-19 has been documented in Europe and which is characterized by a widespread diffusion of the disease since March 2020. The same analysis has been performed for all the other Italian regions and in the Supplementary Information (SI) we present results for other five more populated regions (Lazio, Campania, Sicily, Veneto, Emilia-Romagna).

## Results: the case reproduction number $R_c$ and the optimal generation time distribution

In Fig. 1 we plot $\{I\}$ which clearly shows the presence of two waves in the disease spreading with the peak of infection reached on March 24, 2020, during the first wave, and on November 10, 2020, during the second one. Here we present results assuming that *w*(*z*) is a Gamma distribution, $w(z)=\left( \tau ^{-a}/\Gamma (a) z^{a-1}\right) \exp (-z/\tau )$, which depends on two parameters, $a\ge 1$ and $\tau >0$, and where $\Gamma (a)$ is the Gamma function. The mean value of *w*(*z*) is given by ${{\overline{z}}}=a \tau$ and its standard deviation $\sigma =\sqrt{a} \tau$. In the SI (Figs. Suppl. 4, 5) we show that a log-normal and a Weibull distributed *w*(*z*) produce similar results.

**Figure 2:**
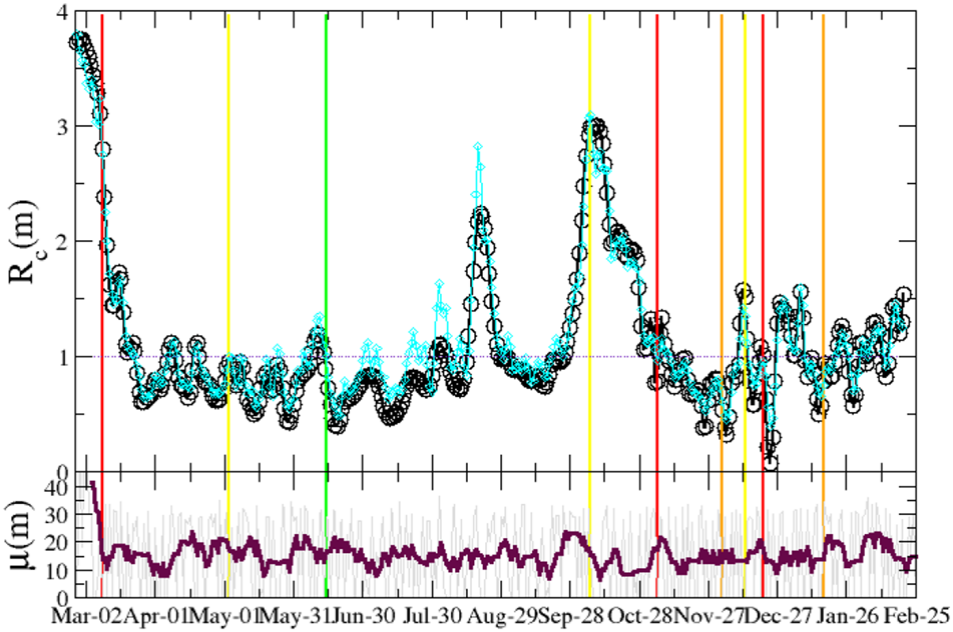
(Upper panel) The case reproduction number $R_c(m)$ of COVID-19 for the Lombardy from 02/24/2020 up to 02/18/2020. Color vertical lines indicate the starting time of different containment measures (see caption of Fig. 1). Black circles represent $R_c(m)$ obtained by means of the log-likelihood maximization procedure whereas cyan diamonds are used for $R_c(m)$ estimated from the WT method [Eq. (5) in “Methods”]. (Lower panel) The daily number of imported cases $\mu (m)$ estimated by the log-likelihood maximization procedure is plotted in thin grey whereas solid line is used for its weekly average.

**Figure 3:**
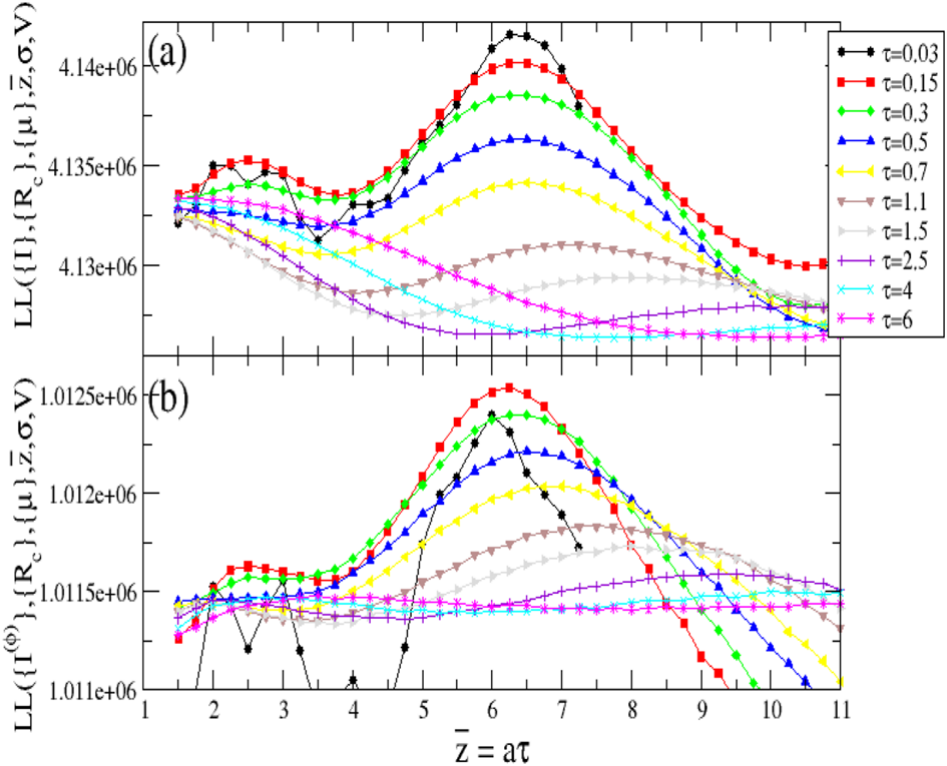
(**a**) The log-likelihood $LL\left( \{I\},\{R_c\},\{\mu \},{{\overline{z}}},\sigma ,V\right)$ obtained from the daily incidence of COVID-19 in Lombardy, is plotted as a function of ${{\overline{z}}}=a \tau$. Different curves correspond to different values of $\tau$, which implies a different $\sigma =a \sqrt{\tau }$. (**b**) As in the upper panel for $LL\left( \{I^{(\phi )}\},\{R_c\},\{\mu \},{{\overline{z}}},\sigma ,V\right)$ with $I^{(\phi )}(m)$ given in Eq. (4) for $\phi =\phi ^*=5E-4$.

We use the data plotted in Fig. 1 to extract the information about $\{R_c\}$, $\{\mu \}$ and *w*(*z*) according to our maximization procedure of $LL\left( \{I\},\{R_c\},\{\mu \},{\overline{z}},\sigma ,V\right)$, exploring in detail a wide range of ${{\overline{z}}}$ and $\sigma$ values. In Fig. 2 we plot the temporal variation of $R_c(m)$ and $\mu (m)$, which have been obtained for ${{\overline{z}}}=6.2$ and $\sigma =0.95$ corresponding to a maximum of *LL* (Fig. 3) and therefore representing an optimal description of the recorded sequence $\{I\}$. In order to verify the efficiency of our procedure we implement these optimal series $\{R_c\}$, together with the optimal choices of ${{\overline{z}}}$ and $\sigma$, in the generation tree algorithm (“Methods”). It is worth noticing that in order to obtain the value of $R_c(m)$ on the *m*-th day it is necessary to know the incidence rate of subsequent days, and a reasonable estimate of $R_c(m)$ is possible only up to the time $m \lesssim N-{\overline{z}}$.

In the algorithm we also implement the optimal series $\{\mu \}$ extracted from the *LL* maximization and therefore only one free arbitrary parameter *I*(0), representing the initial value of infected people on the 0-th day, survives. In Fig. 1 we show that the numerical sequence $\{I\}$ simulated via the generation tree algorithm very well overlaps with the experimental one. As a further support we also compare our findings for $R_c(m)$ with the one provided by the Wallinga–Teunis (WT) algorithm [see Eq. (5) in “Methods”]^18^. We observe (Fig. 2) that the two approaches, for the same *w*(*z*), provide very similar results. Fig. 2 also shows a clear decrease of $R_c(m)$ after the application of strong confinement measures (after red lines) with a weak tendency to an increase after the removal of these measures. In particular, it is interesting to observe a clear peak of $R_s(m)$ in the middle of August which reflects the intense social activity typical of Italian summer vacation at the turn of the Assumption (August 15th). The observed behavior of $R_c(m)$ is therefore consistent with its expected dependence on the contact rate. In the lower panel of Fig. 2 we plot the weekly average value of $\mu (m)$ which fluctuates around an average value $\mu \sim 15$ daily cases. We notice that a clear decrease of $\mu (m)$ from $\mu (m)\simeq 40$ to $\mu (m) \simeq 10$ is observed at March 2020 at the beginning of the recording series (first red line in Fig. 2) in correspondence with the introduction of the first lockdown in Lombardy. A decreasing trend, but much less pronounced (from $\mu (m)\simeq 20$ to $\mu (m) \simeq 10$), is also observed in correspondence with the introduction of the other two lockdowns (second and third red line in Fig. 2). We conversely observe that the release, or the partial release, of lockdown (green lines in Fig. 2) substantially does not affect $\mu (m)$. The origin of this behavior can be attributed to the occurrence of the release when the incidence rate was in a fast decreasing period (Fig. 1). Accordingly, the increase in the mobility of individuals has been substantially balanced by the decrease of the percentage of infected individuals, leading to an about constant $\mu (m)$. There are no other estimates for $\mu (m)$ in Italian regions whereas the number of imported cases has been recorded by the Hong Kong Centre for Health Protection (HKCHP)^35^. Preliminary results indicate a qualitative agreement between our estimate of $\mu (m)$ in Hong Kong and the number of imported cases in the HKCHP dataset. At the same time, we can perform an indirect test of the efficiency of our approach by implementing the value of $\mu (m)$, obtained by our method, as the input parameter of the generation tree algorithm (see “Methods”) which allows us to simulate a synthetic sequence $\{I\}$. In Fig. 1 we show that this synthetic sequence is in excellent agreement with the recorded daily incidence and this represents a strong check supporting the validity of our inversion procedure for both $R_c(m)$ and $\mu (m)$.

An interesting result is provided by Fig. 3a where we plot $LL\left( \{I\},\{R_c\},\{\mu \},{\overline{z}},\sigma ,V\right)$ as function of ${\overline{z}}$. The equivalent of Fig. 3a for a log-normal and for a Weibull distributed *w*(*z*) is presented in Figs. Suppl. 4a and 5a in SI. We find that *LL*, at fixed $\tau$, presents a non-monotonic behavior with two maxima which are more evident for small $\tau$ values. The position ${\overline{z}}$ of the absolute maximum appears to be quite independent of $\tau$. We wish to stress that the presence of local maxima makes very complicated the identification of the optimal *a* and $\tau$ by means of standard methods for *LL* maximization^36^. Automatic procedures can be indeed trapped in a local maximum. Conversely, our fast *LL* evaluation allows us to explore a wide range of *a* and $\tau$ values, except for $\tau <0.03$ which are not numerically accessible because of the divergence of $\Gamma (a)$ when $a\gg 1$. Considering data at fixed ${\overline{z}}$ we notice that *LL* tends to decrease for increasing $\tau$, i.e. increasing $\sigma$. The main result is that the optimal value (maximum value of *LL*) is obtained for ${\overline{z}}=6.2$ days and $\tau =0.03$, corresponding to $\sigma =0.45$ days. A previous estimate ${{\overline{z}}}=6.6$ days was provided for Lombardy from the distribution of serial intervals, extracted from the analysis of contact tracing data^11,12^. We find that the estimates for ${{\overline{z}}}$ are in reasonable agreement whereas important differences are found in the $\sigma$ values. For instance serial intervals in Lombardy give $\sigma =4.9$ days^11,12^ which is much larger than our estimate $\sigma =0.45$ days. We remark that we obtain a similar estimate of $\sigma \simeq 0.5$ days for all 21 Italian Regions and for the different functional forms of *w*(*z*) (see SI).

## How to manage periodicity in data collection

**Figure 4:**
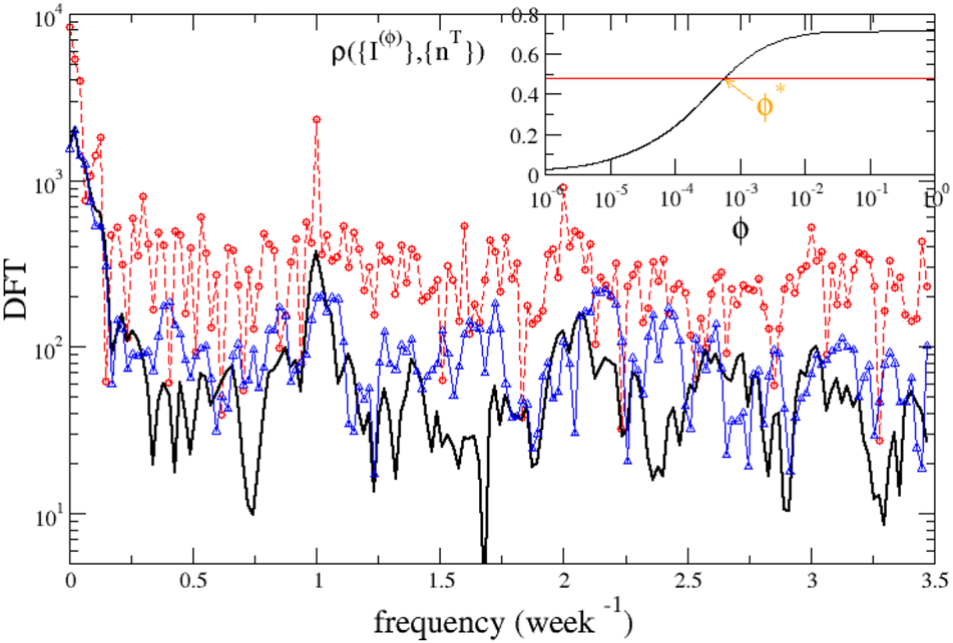
The DFT of the series $\{I\}$ (black lines), $\{n^T\}$ (red circles) and $\{I^*\}$ (blue triangles) as function of the frequency. Time is measured in week units such that the peak in 1 corresponds to a weekly periodicity. (Inset) The Pearson’s correlation coefficient $\rho \left( \{I^{(\phi )}\},\{n_T\}\right)$ as function of $\phi$. The horizontal red line represent the value $\rho \left( \{I^*\},\{n^T\}\right)$.

Figure 3a indicates that the maximum of *LL* is located at the smallest accessible value of $\tau$ ($\tau =0.03$) and therefore does not exclude that a larger *LL* can be obtained for a even smaller value of $\tau$. This would correspond to an even smaller $\sigma$ and, as a consequence, to an even more peaked distribution *w*(*z*). This scenario can be excluded by the behavior of $LL\left( \{I\},\{R_c\},\{\mu \},{\overline{z}},\sigma ,V\right)$ obtained implementing a log-normal distributed *w*(*z*) (Fig. Suppl. 4a). In this case we are able to explore values of $\sigma$ as small as 0.1 days and we find (Fig. Suppl. 4a) that the peak of $LL\left( \{I\},\{R_c\},\{\mu \},{\overline{z}},\sigma ,V\right)$ at ${{\overline{z}}}=6$ is non monotonic as function $\sigma$ with a maximum value for $\sigma =0.45$, in agreement with the results of Fig. 3a. However, in this section we show that the weekly periodicity in the testing procedure can be responsible of a underestimate of the correct $\sigma$ value. The periodicity is caused by the fact that a smaller number of tests are performed during the weekend with respect to the working weekdays and can be clearly enlightened by the daily number of new tested people $\{n^T\}$ whose Discretized Fourier Transform (DFT) presents (Fig. 4) a peak at a frequency value equal to one, when time is measured in week units. The behavior of the DFT of $\{n^T\}$ at small frequencies can be conversely related to the periodicity caused by the two waves. Indeed, an increasing (decreasing) number of infected induces a larger (smaller) number of tests, leading to a correlation between the two signals $\{n^T\}$ and $\{I\}$. The same peak at small frequencies is indeed also found in the DFT of $\{I\}$ where a second smaller peak at $f=1 week^{-1}$ is still observed (Fig. 4). This second peak can be attributed to the weekly periodicity in the number of tests.

In the following we develop a simple argument to disentangle the daily number of infected $\{I\}$ from the daily test number $\{n^T\}$. More precisely we assume that number of identified infected, during the *m*-th day, can be viewed as the sum of two contributions $I(m)=I^{(\phi )}(m)+I^{ran}(m)$. Here $I^{ran}(m)$ represents asymptomatic individuals who are identified as infected, on the *m*-th day, substantially by chance, according to a random search within a population $N_P$. Indicating with $I^{TOT}(m)$ the total number of new infected individuals during the *m*-th day, and taking into account that the search is not fully random but it is usually focused on a subset $N_P \phi _1$ of the total population, we have $I^{ran}(m)=n^T(m) I^{TOT}(m)/(\phi _1 N_P)$, with $\phi _1 <1$. The quantity $I^{(\phi )}(m)$ conversely includes all infected with symptoms and all individuals who have been in strict contact with them. It is reasonable that these individuals are always tested and therefore their identified infection is not related to the daily number of performed tests. We define it as the “disentangled” incidence rate since we expect that its value does not depend on $n_T(m)$. Assuming that $I^{(\phi )}(m)$ is a fixed fraction $\phi _2 < 1$ of the total number of infected, $I^{(\phi )}(m)=\phi _2 I^{TOT}(m)$, we obtain $I(m)=I^{TOT}(m) \phi _2+I^{TOT}(m) n^T(m)/(\phi _1 N_P)$, and therefore the disentangled incidence daily rate $I^{(\phi )}(m)$ can be written as

4 $$\begin{aligned} I^{(\phi )}(m)=\frac{I(m)}{1+\frac{n^T(m)}{\phi N_P}} \end{aligned}$$

where $\phi =\phi _1 \phi _2$ is a parameter which can be fixed by imposing that $\{I^{(\phi )}\}$ is not causally related to $\{n^T\}$. More precisely we observe that (inset of Fig. 4) the Pearson’s correlation coefficient, $\rho \left( \{I^{(\phi )}\},\{n^T\}\right)$, between the temporal series $\{I^{(\phi )}\}$ and $\{n^T\}$ is a monotonic increasing function of $\phi$. We therefore identify the optimal threshold $\phi ^*$ for decorrelation by imposing $\rho \left( \{I^{(\phi )}\},\{n^T\}\right) =\rho \left( \{I^*\},\{n^T\}\right)$. Here $\{I^*\}$ is the temporal series obtained by randomly reshuffling the original series $\{I\}$ in such a way that $I^*(m)=I(m^*)$, where $m^*=m+k^*$ and $k^*$ is an integer random number uniformly distributed in the interval $[-3:3]$. By construction $\{I^*\}$ cannot present the weekly periodicity of $\{I\}$, as confirmed by its DFT (Fig. 4). The condition $\rho \left( \{I^{(\phi )}\},\{n^T\}\right) =\rho \left( \{I^*\},\{n^T\}\right)$, therefore, allows us to obtain, after setting $\phi =\phi ^*$ in Eq. (4), a series $\{I^{(\phi )}\}$ whose daily variation is uncorrelated to the weekly periodicity of $\{n^T\}$. In Fig. 3b we plot $LL^{(\phi )}=LL\left( \{I^{(\phi )}\},\{R_s\},\{\mu \},{{\overline{z}}}, \sigma ,V\right)$ as function of ${{\overline{z}}}$, for different values of $\tau$ and $\phi =\phi ^*$. This figure still shows the presence of a maximum at ${{\overline{z}}}=6.1$ and interestingly, at fixed ${\overline{z}}$, $LL^{(\phi )}$ non monotonically depends on $\tau$, with the largest value reached for $\tau =0.15$. This leads to $\sigma =0.95$ days which represents a more reasonable estimate than the smaller value suggested by Fig. 3a. Similar results are obtained for similar choices of $\phi \in (0.2 \phi ^*, 5 \phi ^*)$, for different functional form of *w*(*z*) and also for the majority of Italian regions (see SI) even if, in few cases, the value of $\phi ^*$ is so small to hide the information contained in $\{I^{(\phi )}\}$ leading to a less significant $LL^{(\phi )}$ (see Fig. Suppl. 11d, as an example).

We remark that the disentangled incidence rate $\{I^{(\phi )}\}$ also allows us to take better into account the incidence of asymptomatic people that remain undetected. Their contribution can be neglected in the hypothesis that their percentage remains constant over time, whereas their temporal variations could introduce a wrong estimate of the transmission parameters. Nevertheless, since the quantity $\{I^{(\phi )}\}$ should represent all individuals which are certainly tested it is expected to be much less affected by the contribution of asymptomatic undetected people with respect to the original incidence rate $\{I\}$. Accordingly, the observation that the two incidence rates $\{I^{(\phi )}\}$ and $\{I\}$ lead to similar results indicates that our procedure is only weakly affected by temporal variations in the percentage of asymptomatic people. To further support this conclusion, in Suppl. Fig. 8 we present results using for the incidence rate the daily number of individuals entering the intensive care units $I^{ICU}$. We obtain results that do not differ significantly from the one obtained for $\{I^{(\phi )}\}$, further supporting the stability of our findings. In Suppl. Fig. 3 we also show that a similar estimate of the optimal ${\overline{z}}$ and $\tau$ value is obtained by applying a Butterworth filter to *I*(*m*) to filter out Fourier components in the frequency range $[1/7.5,1/6.5] days^{-1}$.

## Optimal quarantine duration & fast switching protocols

Using the best estimate ${\overline{z}}=6.1$ and $\sigma =0.95$ we obtain that the residual risk of virus transmission after a quarantine period of 10 days is smaller than $0.05 \%$. Our estimate is smaller than the one considered by the Centers for Disease Control and Prevention (CDCP) which indicates that at Day 10, with symptom monitoring but without diagnostic testing, the estimated residual post-quarantine transmission risk is $1.4\%$ with a range $[ 0.1\%-10.6\%]$. We remark that this estimate is mostly based on the model proposed in Ref.^37^ which uses as a key information the serial interval distribution obtained in ref.^27^. As already shown in Ref.^15^, the estimate of $\sigma _s$ obtained by the serial interval analysis of Ref.^27^ is significantly larger than the standard deviation $\sigma$ of generation time and this can be responsible for an overestimation of the post quarantine transmission risk. In order to better explore the effect of $\sigma$ on the optimal quarantine duration $t_q$, we consider the numerical model [Eq. (2)] under the assumption that individuals which have been in contact with an infected person can be put in isolation for a quarantine period $t_q$. More precisely, we consider a constant number of daily imported cases $\mu (m)=10$, a constant reproduction number $R_t(t)=R_0=3$ and a mean value of the generation time ${\overline{z}}=6$ days. We then assume that a fraction $\xi$, randomly selected within the population of infected individuals, are put in quarantine. This corresponds to remove from the infectious tree all the secondary cases infected by a primary case during his quarantine period. To evaluate the efficiency of the quarantine policy, for different values of $\xi$, we measure the effective exponent $\alpha =\log \left( \frac{I(t_f)}{I(t_0)}\right)$ with $t_f=350$ days and $t_0=100$ days. The exponent $\alpha$ controls the slope of the epidemic curve and, for instance, a value $\alpha =0$ indicates a constant *I*(*t*) whereas in the case of an exponential growth of *I*(*t*), $\alpha$ becomes much larger than 1.

**Figure 5:**
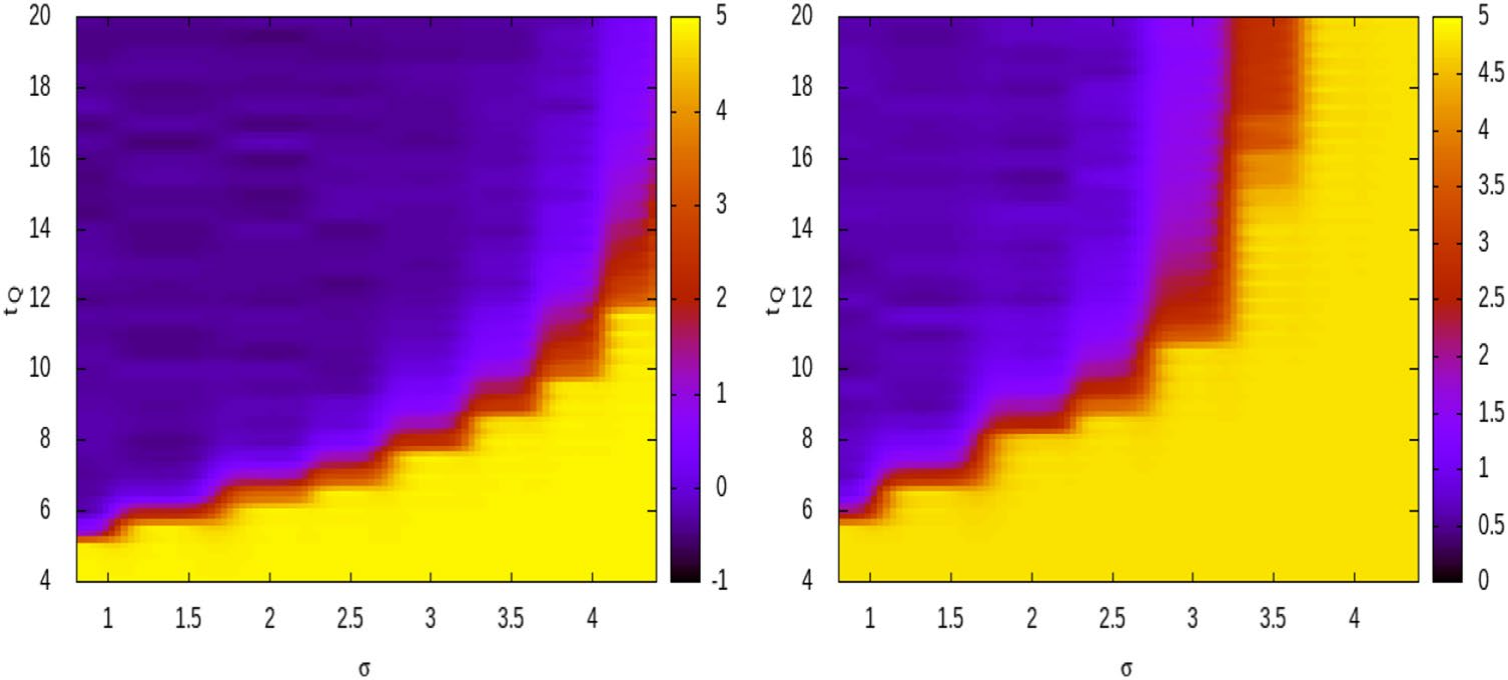
The contour plot of the effective exponent $\alpha$ which controls the growth velocity of the disease is plotted for different $\sigma$ and different duration of the quarantine period $t_Q$, for numerical simulated *I*(*t*) with $\mu _m=10$ and $R_c(m)=3$ and a precentage $\xi$ of population put in quarantine. Left panel presents results for $\xi =0.75$ whereas right panel for $\xi =0.66$. We assume that *w*(*z*) is Gamma distributed with ${\overline{z}}=6.1$ days.

The contour plot of $\alpha$ as function of $\sigma$ and $t_q$ is presented in Fig. 5 which shows that, for all values of $\xi$, there exists a transition between the smaller $\alpha$ from the larger $\alpha \gtrsim 1$ region and therefore it is easy to identify the optimal duration of the quarantine period $t_q^{opt}$ such as for $t_q>t_q^{opt}$ one is always in the $\alpha \lesssim 1$ region where the virus spreading is under control. We find that $t_q^{opt}$ is an increasing function of $\sigma$, as expected, and for $\xi \le 2/3$ no quarantine option appears efficient in spreading reduction if $\sigma \gtrsim 4$.

**Figure 6:**
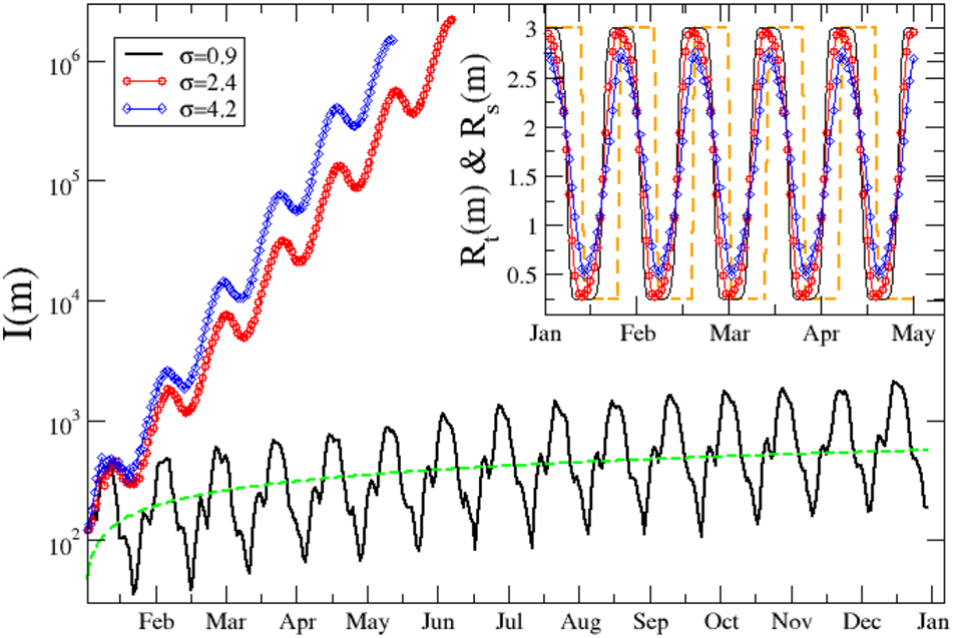
The simulated daily incidence *I*(*m*) for a periodic fast switching protocol which alternates period without constrains to rigid lockdown periods, each one lasting 12 days. We assume $I(0)=1000$ at the starting time. Different colors and symbols are used for different values of $\sigma$ in the probability distribution of generation time *w*(*z*), which is Gamma distributed. The green dashed line is a power law fit $m^{1/2}$ for the temporal evolution of the average value of *I*(*m*) when $\sigma =0.95$. (Inset) We plot with dashed orange line the evolution of $R_t(m)$ implemented in the numerical model. The other curves represent the evolution of $R_c(m)$ for different values of $\tau$, with the same color code and symbols of the main panel.

We next discuss the influence of $\sigma$ in fast periodic switching protocols^38^ where periods of stringent lockdown alternate with periods when only weak social constraints are imposed. The same consideration applies to the protocol of alternating quarantine^39^, where the population is subdivided in two non interacting subsets, each one subject to a fast periodic switching protocol in phase opposition: While one subset is in full lockdown, the other subset has regular activity. These protocols have the advantage to suppress the virus outbreak, while at the same time they allow for continued economic activities. More precisely, we take as reference value $R_c(m)=3$ measured in Lombardy at the beginning of October 2020, in a temporal period where substantially all the activities, including schools, were open. We therefore assume that during an interval of duration $T_{NLD}$ the reproduction number $R_t(m)$ assumes the constant value $R_t(m)=3$, whereas in the subsequent period of duration $T_{LD}$ a rigid lockdown decreases $R_t(m)$ to very low values. It is quite intuitive that in order to quench existing infections, it is necessary that $T_{LD} > {{\overline{z}}}+ \sigma$, so that an infected individual will have a low probability to be still infectious when s/he comes back to normal activity. To test this point we simulate the evolution of *I*(*m*) over a temporal interval of one year, assuming a periodic $R_t(m)$ which alternates, each $T_{LD}=T_{NLD}=2 {{\overline{z}}}$, between the values of $R_t(m)=3$ and $R_t(m)=0.25$, leading to a periodic $R_c(m)$ (inset of Fig. 6) which, according to Eq. (1) follows $R_t(m)$ with a delay which depends on $\sigma$. We always assume a Gamma distributed *w*(*z*) with ${{\overline{z}}}=6.1$ days, obtained from our analysis, and we consider two values of $\sigma$, $\sigma =0.95$ obtained from our analysis and $\sigma =4.9$ days estimated from serial intervals^11,12^. Results (Fig. 6) clearly show that for a sufficiently large $\sigma$, *I*(*m*) presents fluctuations around an average exponential growth which is steeper for larger $\sigma$. Obviously, in this case the fast switching protocol does not work. Conversely, for $\sigma =0.95$ the exponential growth is replaced by a much slower power law increase and *I*(*m*) fluctuates around an average value which changes very slowly in time. We can finally conclude that only in the case of small $\sigma$ fast switching strategies give the possibility to keep the disease spreading always under control leaving simultaneously enough space to normal activities.

## Discussion and conclusions

We have presented a detailed analysis of the log-likelihood $LL\left( \{I\},\{R_c\},\{\mu _m\},{{\overline{z}}}, \sigma ,V\right)$ for the daily incidence rate $\{I\}$ of COVID-19 in different Italian regions. We have presented a new method which provides, for a fixed functional form of the generation time distribution *w*(*z*), the daily evolution of the case reproduction number $R_c(m)$ together with the daily number of imported infections $\mu (m)$. The optimization method is sufficiently fast to allow us to explore a wide range of the parameters ${\overline{z}}$ and $\sigma$, controlling the functional form of *w*(*z*). In this way we are able to estimate the optimal value for all transmission parameters which appear in the renewal equation (Eq. 2). In particular we obtain both the daily evolution of the reproduction number but also the optimal functional form of *w*(*z*). The efficiency of the method is demonstrated by the fact that implementing these optimal values in the numerical code we find a numerical daily incidence rate which is in very good agreement with the experimental one. The achieved information can be used to test and optimize control strategies to mitigate the damage via the identification of the key parameters ${\overline{z}}$ and $\sigma$ which can be obtained in a learning period $m \in [1,m_{learning}]$. Interestingly, when we consider $m_{learning}=10$ days or $m_{learning}=30$ days, we already find (see Fig. Suppl. 6) the optimal values ${\overline{z}}= 6.1$ and $\sigma =0.95$, in agreement with results obtained when $m_{learning}=1$ year. This indicates that we can have a good estimate for the generation time distribution already after 11 days from the starting of data collection. Other methods^13–15^ based on contact tracing, conversely, needs a longer temporal window to collect the correct infector-infectee pairs and to have sufficient data to measure the incubation period distribution. The stability of the optimal values of ${\overline{z}}$ and $\sigma$, for different learning period, also indicates that their estimate appears only weakly influenced by the enhancement of NPI. which conversely strongly affects^16,17^ the evaluation of the mean serial interval obtained via contact tracing.

In particular, the small value of $\sigma \simeq 1$ shows that an individual remains infectious in a small temporal window between $\sim [4:8]$ days, from the starting time of his/her infection, and transmission risk is very small outside this interval. This information can be useful to better characterize the biological processes promoting the transmission of the disease and in planning optimized strategies for mitigating the spreading of the virus. For instance, our study indicates that it is sufficient a quarantine period no longer than 8 days. Moreover, we have shown that it could be possible to keep the number of daily infected quite constant by means of a fast periodic switching protocol which alternates periods of 12 days of rigid lock-down with unconstrained periods. In this way it is possible to avoid the exponential growth of the virus spreading while keeping socio-economic activity ongoing at about the $50\%$ of maximum capacity.

## Methods

### The generation tree algorithm

We present a standard procedure for a self-exciting branching process where the time series *I*(*m*) is simulated according to a generation tree algorithm^40^. The first step is setting the number *I*(0) of infected persons on the day 0 and use the daily number of imported infections $\mu (m)$ to obtain $n_0=I(0)+\sum _{m=1}^N\mu (m)$, the number of infected people. Using the terminology of branching processes, this is the zero-th order generation and we index with $k_0\in [1,n_0]$ each infector, defined as mother element, which generates a number $n(k_0)$ of off-springs, i.e. the newly infected elements. The number $n(k_0)$ of off-springs depends on $R_c(t(k_0))$ evaluated at the occurrence time $t(k_0)$ of their mother, according to a Poisson distribution $P_P(n(k_0))$ with the expected value $E(n(k_{0}))=R_c(t(k_0))$. This is the first order generation containing $n_1=\sum _ {k_0=1}^{n_0}n(k_0)$ elements, each one infected at a time $t(k_1)=t(k_0)+z$, where *z* is a random time extracted according to the probability generating function *w*(*z*). Only off-springs infected during the observational time window $t(k_1) \in [1,N]$ are considered. At the subsequent step $(j+1)$ the previous step is repeated considering as mother elements the $n_j$ off-springs of the previous $(j-1)$ generation. In this way one obtains $n_{j+1}$ new off-springs and the process is iterated up to the final generation $j_f$, such that $n_{j_f+1}=0$. The numerical code is available for open access at https://github.com/Statistical-Mechanics-Group-Caserta/covid-maximum-loglikelihood-estimation.

### The log-likelihood maximization procedure

The algorithm assumes an initial trial value of $\{R_c^{0}\}$ and $\{\mu ^0\}$. At each Monte-Carlo (MC) trial we randomly select a day $m'\in [1,N]$ and extract $\delta R= q_r r R_s(m')$ with *r* uniformly distributed between $[-1/2,1/2]$ and $q_r \ll 1$. We evaluate *E*[*I*(*m*)] replacing in Eq. (2) $R_c(m')=R_c(m')+\delta R$. The new value of *E*[*I*(*m*)] is used in Eq. (3) for the evaluation of the trial log-likelihood $LL'$. If $LL'>LL$ the new value of $R_c(m')$ is more consistent with data and its value is therefore retained, otherwise it is discarded. A similar procedure is applied to the series $\{\mu \}$ with the trial value $\mu (m')=\mu (m')+q_{\mu } r$ (with $q_\mu \ll 1$) accepted only if it leads to a larger value of $LL'>LL$ from Eq. (3). We complete a Monte-Carlo step when *N* trials have been performed. The new value $R_c(m')$ only affects terms with $m>m'$ in the first sum in the rhs of Eq. (3) and therefore the number of operation in a MC step is of order $N\times N/2$ making the computation very fast with 5000 MC steps involving about 10 s of standard CPU time, when $N=360$ days. Since $\{\mu \}$ only weakly affects *LL*, the evaluation is optimized by including the trials on $\{\mu \}$ only each 20 MC steps. We have verified that simulations evolve towards an asymptotic value which is independent of the specific initial choice of $\{R_c^{0}\}$ and $\{\mu ^0\}$. Indeed, only the initial value $R_c^0(0)$ is relevant since it remains constant during the simulation and therefore affects the other values of $R_c(m)$, because of the smoothness constraint (Eq. 3). For this reason we extract this value by means of the Wallinga-Teunis (WT) method^18^ which, from Eq. (1), can be written as $R_c(m)=\sum _{j=m}^N\beta (j,m)$, leading to

5 $$\begin{aligned} R_c(j)=\sum _{m=0}^N \frac{I(j+m)w(j)}{\sum _{l=0}^N I(j+l-m)w(l) }, \end{aligned}$$

which is obtained implementing the definition $\beta (m,j)=R_t(m) w(m-j)$ and using $R_t(m)=E(I(m))/ \sum _{j=1}^N w(m-j) I(j)$, given by Eq. 2 after setting $\mu (m)=0$. In particular, we use the initial value of $R_c^{0}(m)$ from Eq. (5) for $m\le 3$, whereas for $m\ge 4$ we assume that $R_c^0(m)$ linearly decreases to $R_c^0(m)=0.1$ and then remains constant at larger *m*. We also fix $\mu ^0(m)=0$ for all $m\in [1,N]$. Results do not depend on this initial choice. Concerning the value of the parameter *V*, from Eq. (1), we have that significant changes of $R_c(s)$ are possible only on time scales larger than $\sigma$ and we have verified that setting $V=3.5/\sqrt{\sum _{m=1}^NI(m)}$ in Eq. (3) this condition is satisfied.

We have verified that after 5000 MC steps the simulation reaches its asymptotic value, namely $\{R_c\}$ and $\{\mu \}$ do not vary appreciably for additional MC steps. The numerical code is available for open access at https://github.com/Statistical-Mechanics-Group-Caserta/covid-maximum-loglikelihood-estimation.

### The independence between *w*(*z*) and $\Delta t_{rec}$

We indicate with $I^{(true)}(m)$ the number of infections which truly occurred on the *m*-th day. This infection, however, is identified and reported only subsequent days. More precisely, we indicate with $\psi (\Delta t_{rec})$ the distribution of the time delay $\Delta t_{rec}$ between the time of the infection and the time when this infection is identified and reported in the data set. Accordingly, the daily rate of recorded infections can be written as

6 $$\begin{aligned} I(m)=\sum _{n=-\infty }^{\infty }I^{(true)}(n) \psi (m-n) \end{aligned}$$

where we extended the sum over *n* to the range $(-\infty ,\infty )$ assuming that $I^{(true)}(m)=0$ if *m* is smaller than the first detection day and also $\psi (j)=0$ if $j\le 0$. We have also assumed that $\psi$ is a stationary distribution which depends only on the time difference $m-n$. The renewal Eq. (2) is expected to hold for $I^{(true)}(m)$

7 $$\begin{aligned} E[I^{(true)}(m)]=\sum _{j=-\infty }^{m-1}R_c(j) w(m-j)I^{(true)}(j) +\mu (m) \end{aligned}$$

but we will show that it also holds for *I*(*m*). We first introduce the quantity ${\tilde{w}}(k)=w(k)$ if $k > 1$ and ${\tilde{w}}(k)=0$ if $k \le 1$ which allows us to write Eq. (7) as

8 $$\begin{aligned} E[I^{(true)}(m)]=\sum _{j=-\infty }^{\infty }R_c(j) {\tilde{w}}(m-j)I^{(true)}(j) +\mu (m). \end{aligned}$$

Inserting Eqs. (8) in (6) we obtain

9 $$\begin{aligned} E[I(m)]=\sum _{n=-\infty }^{\infty } \sum _{j=-\infty }^{\infty } \left( R_c(j) {\tilde{w}}(n-j) I^{(true)}(j) +\mu (n) \right) \psi (m-n). \end{aligned}$$

that after the change of variables $k=n-j$ can be rewritten as

10 $$\begin{aligned} E[I(m)]=\sum _{k=-\infty }^{\infty } {\tilde{w}}(k) \sum _{j=-\infty }^{\infty } R_c(j) I^{(true)}(j) \psi (m-k-j)+ \mu ^{(rec)}(m), \end{aligned}$$

with $\mu ^{rec}(m)=\sum _{n=-\infty }^{\infty } \mu (n)\psi (m-n)$. We next assume that the time evolution of $R_c(j)$ is sufficiently slow to be considered roughly constant during the time scale where $\psi (m-j-k)$ goes to zero

11 $$ \sum _{j=-\infty }^{\infty } R_c(j) I^{(true)}(j)\psi (m-k-j) \simeq R_c(m-k) \sum _{j=-\infty }^{\infty } I^{(true)}(j)\psi (m-k-j) = R_c(m-k) I(m-k), $$

where in the last step we used Eq. (6). We finally obtain

12 $$\begin{aligned} E[I(m)]\simeq \sum _{k=-\infty }^{\infty } R_c(k) {\tilde{w}}(m-k) I(k) +\mu ^{rec}(m)=\sum _{k=-\infty }^{m-1} R_c(k) w(m-k) I(k) +\mu ^{rec}(m) \end{aligned}$$

which is the renewal equation for *I*(*m*) no longer depending on $\psi (\Delta t_{rec})$. In particular the generation time distribution *w*(*z*) obtained from Eq. (12), for the daily recorded incidence rate *I*(*m*), is the same controlling the evolution of $I^{(true)}(m)$ [Eq. (7)].

## Supplementary Information

Supplementary Information.

## Data Availability

We consider data provided by Protezione Civile for the 21 Italian regions and collected in https://github.com/DavideMagno/ItalianCovidData. We consider the time series from February 24, 2020 $(m=1)$ up to February 24, 2021 $(m=N)$ for global $N=366$ days.
