# Supplementary material for: Estimating the generation interval from the incidence rate, the optimal quarantine duration and the efficiency of fast switching periodic protocols for COVID-19
Source: Sci Rep. 2022 Mar 17;12:4623. doi: 10.1038/s41598-022-08197-x (PMC8929281; doi:10.1038/s41598-022-08197-x)
Supplement: Supplementary file 1 — Supplementary Information. [file 41598_2022_8197_MOESM1_ESM.pdf]

SUPPLEMENTARY INFORMATION:Estimating the generation interval from the incidence rate, the optimal quarantine duration and the efficiency of fast switching periodic protocols for COVID-19

E. Lippiello and G. Petrillo
Department of Mathematics and Physics, University of Campania "Luigi Vanvitelli", 81100, Caserta, Italy

L. de Arcangelis
Department of Engineering, University of Campania "Luigi Vanvitelli", 81031, Aversa, Italy

NUMERICAL TEST OF THE RELATIONSHIP BETWEEN $\{I\}$ AND $\{I^{(\phi)}\}$.

In the main text we have introduced the disentangled incidence rate $I^{(\phi)}(m)$ defined as in Eq.(4) of the main text

$$I^{(\phi)}(m) = \frac{I(m)}{1 + \frac{n^T(m)}{\phi N_p}} \tag{1}$$

which for $\phi = \phi^*$ is decorrelated from the daily number of tests $n^T(m)$. We have found that the generation time distribution $w(z)$ obtained from the maximization of $LL^{(\phi)} = LL\left(\{I^{(\phi)}(\phi)\}, \{R_c\}, \{\mu\}, \overline{z}, \sigma, V\right)$ is very similar to the one obtained from $LL\left(\{I\}, \{R_c\}, \{\mu\}, \overline{z}, \sigma, V\right)$. To support our result of a very peaked distribution $w(z)$ we here presents a numerical check which is substantially based on the inversion of Eq.(1). Our approach consists in generating the numerical time series $\{I^{(\phi)}\}$ implementing in our numerical procedure (see Methods in the main text) the values of $\{R_c\}$ and $\{\mu\}$ and a corresponding to the maximum of $LL^{(\phi)}$, for $\tau = 0.15$. We have only one free parameter which is the initial number of infected individuals and setting this number to $I^{(\phi)}(0) = 250$ we find that the numerically simulated $I^{(\phi)}(m)$ (open magenta diamonds in Fig.Supp.1a) is in very good agreement with the recorded one (green curve in Fig.Supp.1a). We next combine the numerical $\{I^{(\phi)}\}$ with the experimental value of $\{n^T\}$ to simulate the total number of detected infected individuals $I(m) = I^{(\phi)}(m)\left(1 + \frac{n^T(m)}{\phi N_p}\right)$, from Eq.(1). We find a very good agreement between the simulated $I(m)$ (filled magenta triangles in Fig.(Supp.1a)) and the recorded one (black continuous line in Fig.(Supp.1a)). Interestingly we find that this agreement is even better than the one obtained in Fig.1 of the main text, with the numerically simulated signal which fits the fluctuations of the recorded one. Furthermore, we also find that the log-likelihood estimated for the numerical series, plotted in Fig.Supp.2a presents a dependence on a and τ very similar to the one observed in Fig.3a of the main text for the experimental one. These results support our decomposition between $\{I\}$ which depends on $\{n^T\}$ and $\{I^{(\phi)}\}$ which conversely is independent of the number of tests.

We next consider numerical simulations still considering a mean value $\overline{z} = 6.2$ days but with a larger $\sigma = 4.2$ days. We find that for any choice of $I^{(\phi)}(0)$, implemented in the generation tree algorithm, it was never possible to generate a numerical sequence $\{I^{(\phi)}\}$ in good agreement with the experimental one. The best numerical $I^{(\phi)}(m)$ (open blue diamonds in Fig.Supp.1b) is clearly different from the experimental one (green continuous lines). Conversely, the agreement significantly improves if we simulate the series $I'(m) = I^{(\phi)}(m)\left(1 + \frac{n^T(m)}{\phi' N_p}\right)$, from the numerically generated $\{I^{(\phi)}\}$, by properly tuning the parameter ϕ'. Fig.Supp.1b indeed shows that $I(m)$ for $\tau = 3.0$ (filled blue triangles in Fig.Supp.1b) follows the experimental curve (continuous black line) even if it is not able to fully capture all the daily fluctuations. The log-likelihood $LL' = LL\left(\{I'_m\}, \{R_c\}, \{\mu\}, a, \tau, V\right)$ however exhibits a functional dependence on a and τ very different from the experimental one. A maximum at $\overline{z} \simeq 6$ is still observed at small τ, but it is not the dominant one. This difference suggests that the behavior of LL observed in instrumental data is difficult to be explained by Eq.(1), unless one does not implement a sufficiently small value of σ in $w(z)$. This further supports the hypothesis that the true probability distribution of generation times $w(z)$ is a very peaked distribution.

REMOVAL OF THE WEEKLY PERIODICITY BY FOURIER FILTERING

We define I^F the signal of the daily incidence of COVID-19 in Lombardy $I(m)$ filtered after the application of a Butterworth filter in the frequency range $[0, 1/7.5[\cup]1/6.5, 1/365]days^{-1}$. The corresponding log-likelihood $LL\left(\{I^F\}, \{R_c\}, \{\mu\}, \overline{z}, \sigma, V\right)$ is plotted in Fig.Supp.3 as a function of $\overline{z} = a\tau$, for different values of τ, which correspond to different values of $\sigma = \tau\overline{z}$. Results show that the dominant peak is observed for $\tau = 0.1$ and $\overline{z} = 6$ in agreement with the results for the log-likelihood of the disentangled signal $LL^{(\phi)}$ (Fig.3b of the main text).

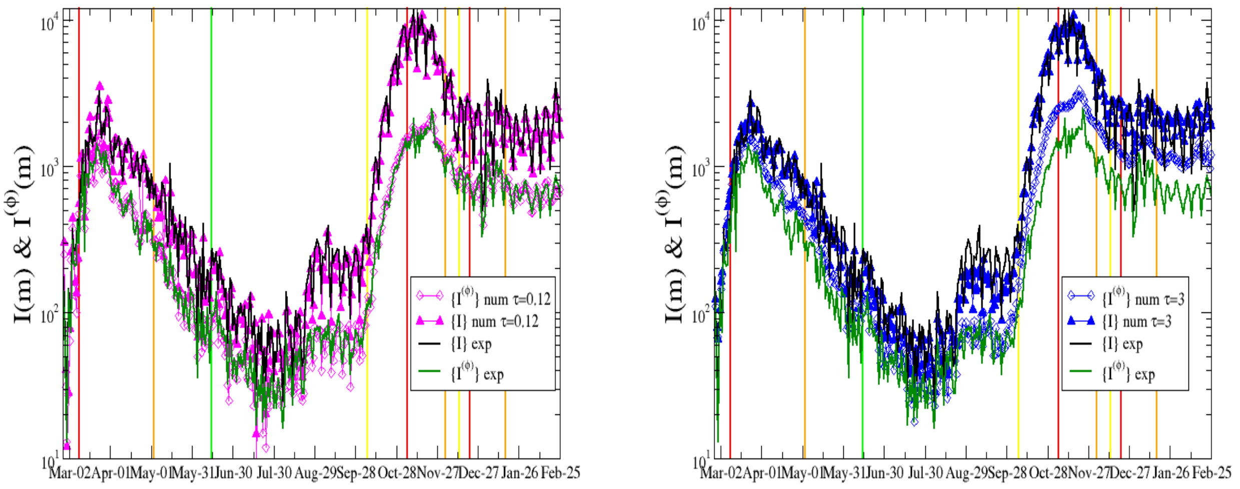

FIG. Supp.1: (Left panel) The daily incidence of COVID-19 $I(m)$ (continuous black lines) and the effective incidence $I^{(\phi)}(m)$ (continuous green lines) in Lombardy are compared with the numerically generated $I^{(\phi)}(m)$ (open magenta diamonds) and $I(m) = I^{(\phi)}(m)\left(1 + \frac{n^T(m)}{\phi N_p}\right)$ (filled magenta triangles), with $\tau = 0.15$ ($\sigma = 0.95$ days) and $\phi = \phi^* = 5E - 3$. We use $I(0) = 250$. (Right panel) The same as for the left panel with numerical data generated with $\tau = 3.0$ ($\sigma = 4.2$ days) for $I^{(\phi)}(m)$ (open blue diamonds) and $I'(m) = I^{(\phi)}(m)\left(1 + \frac{n^T(m)}{\phi' N_p}\right)$ (filled blue triangles). We use $I^{(\phi)}(0) = 100$ and $\phi' = 1E - 3$.

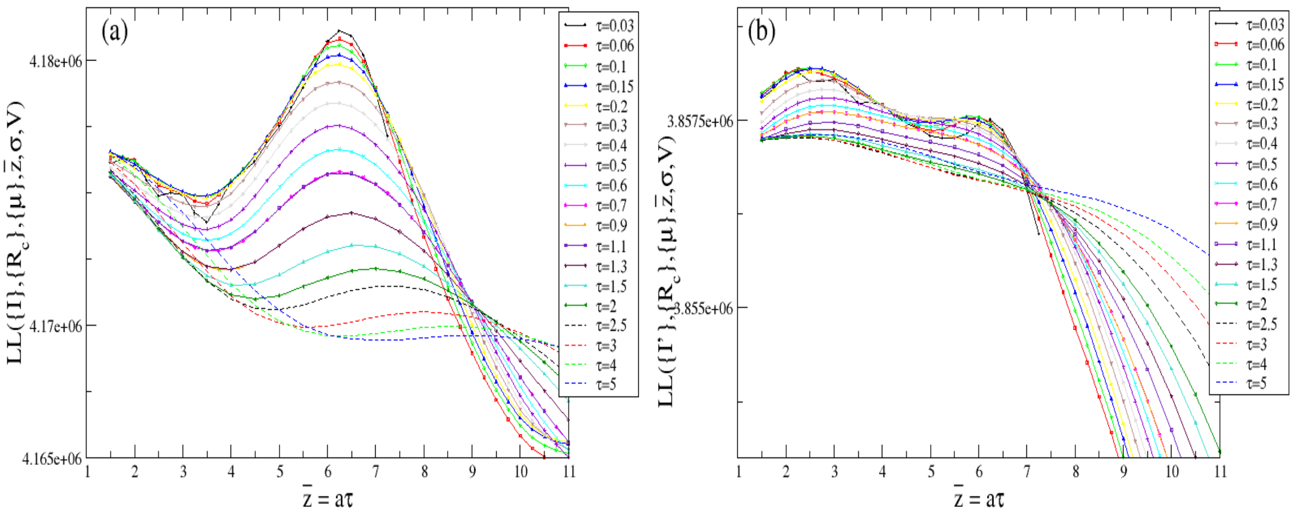

FIG. Supp.2: (Left panel) The log-likelihood $LL(\{I\}, \{R_c\}, \{\mu\}, \overline{z}, \sigma, V)$ where $\{I\}$ represents the numerically generated series implementing the best estimate for $\{R_c\}$ and $\{\mu\}$, for $\tau = 0.15$ and $\overline{z} = 6.0$ days, and plotted in Fig.Supp.1a. (Right pane) The same of the left panel for the numerical series $\{I'\}$ obtained setting $\tau = 3.0$ and plotted in Fig.Supp.1b.

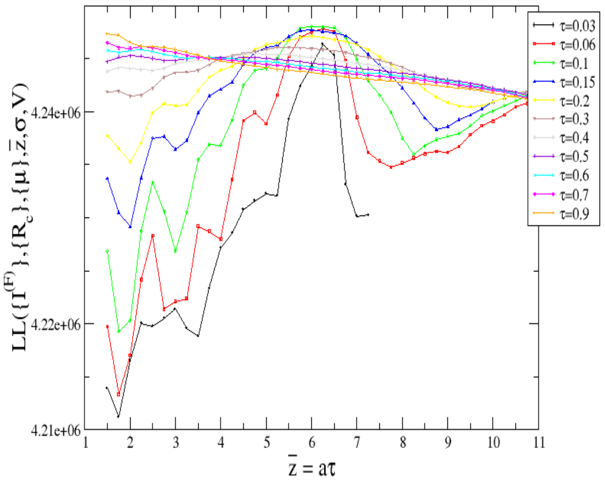

FIG. Supp.3: The log-likelihood $LL\left(\{I^F\},\{R_c\},\{\mu\},\overline{z},\sigma,V\right)$ is plotted as a function of $\overline{z}$. Different curves correspond to different values of τ (see legend).

I. INVERTING BY IMPLEMENTING A LOG-NORMAL AND A WEIBULL DISTRBUTED $w(z)$

In Fig.3a of the main text we present the dependence of $LL\left(\{I\},\{R_c\},\{\mu\},\overline{\tau},\sigma,V\right)$, for the optimal series $\{R_c\}$ and $\{\mu\}$ and different choices of the parameter a and τ of the Gamma distributed $w(z)$. In Fig. 3b of the main text the same analysis was presented for $LL\left(\{I^{(\phi)}\},\{R_c\},\{\mu\},\overline{\tau},\sigma,V\right)$. In this section we present exactly the same study but we assume that $w(z)$ obeys either a log-normal ($w(z)=\frac{k}{\sqrt{2\pi}\lambda z}e^{-\frac{(\log(z)-\mu)^2}{2\lambda^2}}$) or a Weibull distribution ($w(z)=\frac{\mu}{\lambda}\left(\frac{z}{\lambda}\right)^{\mu-1}e^{-\left(\frac{z}{\lambda}\right)^{\mu}}$). The dependence of both functions on two parameters μ,λ can be completely reformulated in terms of the average value $\overline{z}$ and standard deviation. In particular one has $\overline{z}=e^{\mu+\lambda^2/2}$ and $\sigma=\overline{z}\left(e^{\lambda^2}-1\right)$ for the log-normal distribution, whereas $\overline{z}=\lambda\Gamma(1+1/\mu)$ and standard deviation $\sigma=\lambda\sqrt{\Gamma(1+2/\mu)-\Gamma(1+1/\mu)^2}$ for the Weibull distribution. The optimal $LL\left(\{I\},\{R_c\},\{\mu\},\overline{\tau},\sigma,V\right)$ as function of $\overline{z}$ and different λ values is plotted in Fig.Supp.4a and Fig.Supp.5a for the log-normal and the Weibull distribution, respectively. For a better comparison in the same figure we also present data of Fig.3a of the main text for $LL\left(\{I\},\{R_c\},\{\mu\},\overline{\tau},\sigma,V\right)$ in the case of a Gamma distributed $w(z)$ for the smallest values of τ. In the case of the log-normal distribution, $LL\left(\{I\},\{R_c\},\{\mu\},\overline{\tau},\sigma,V\right)$ as function of $\overline{z}$ is very similar to the one find for the Gamma distribution. In particular we obtain, for the Gamma and the log-normal distribution, substantially the same value of the maximum in correspondence to the same value of $\overline{z}$ and σ. Interestingly, with the log-normal distribution we are able to explore smaller values of σ, respect to the minimum one considered for the Gamma distribution, and we find that the maximum of $LL\left(\{I\},\{R_c\},\{\mu\},\overline{\tau},\sigma,V\right)$ is non monotonic with σ but present smaller values when σ values smaller than 0.5 are considered. In the case of the Weibull distribution (Fig.Supp.5a) the comparison is less direct since, at fixed λ the average value $\overline{z}$ is non monotonic with μ. Nevertheless, we still find that the maximum value of LL for $\overline{z}=6.2$ and $\sigma=0.45$ in agreement with the result obtained by a Gamma or a log-normal distributed $w(z)$. In this case the value of $LL\left(\{I\},\{R_c\},\{\mu\},\overline{\tau},\sigma,V\right)$ at the maximum is smaller but still very similar to the one obtained for the Gamma and the log-normal distributed $w(z)$.

The same considerations apply to the behavior $LL\left(\{I^{(\phi)}\},\{R_c\},\{\mu\},\overline{\tau},\sigma,V\right)$ plotted in Fig.Supp.4b for the log-normal distribution and in Fig.Supp.5b for the Weibull distribution. Also in this case we can conclude that no significant differences can be observed between the Gamma and the log-normal distribution with $LL\left(\{I^{(\phi)}\},\{R_c\},\{\mu\},\overline{\tau},\sigma,V\right)$ presenting a very similar dependence on $\overline{z}$ when the same value of σ is imposed in both distribution. Concerning the comparison with the Weibull distribution, we again find a smaller but comparable value of maximum of $LL\left(\{I^{(\phi)}\},\{R_c\},\{\mu\},\overline{\tau},\sigma,V\right)$ with optimal values for $\overline{z}$ and σ very similar to those found for the Gamma and the log-normal distribution. We can therefore conclude that there is no important difference between a Gamma and a log-normal or a Weibull distributed $w(z)$ which all lead to very similar optimal values for $\overline{z}$ and σ.

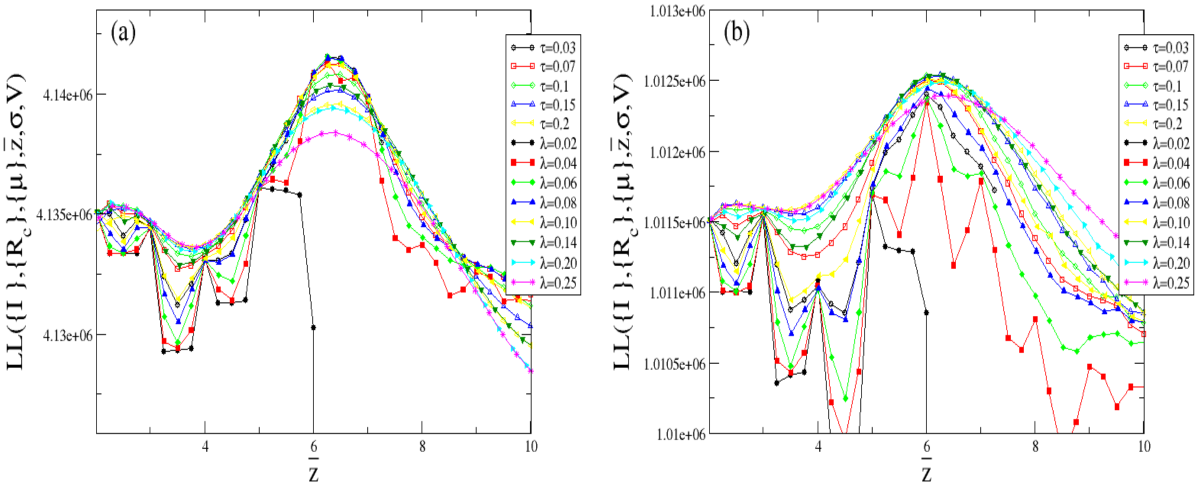

FIG. Supp.4: (Panel a) The log-likelihood $LL\left(\{I\},\{R_c\},\{\mu\},\overline{z},\sigma,V\right)$ for the optimal series $\{R_c\}$ and $\{\mu\}$, for a log-normal distributed $w(z)$ (filled symbols) and a Gamma distributed $w(z)$ (open symbols). Different curves correspond to different values of λ in the case of the log-normal distributed $w(z)$ and to different values of τ in the case of the Gamma distributed $w(z)$, as reported in the legend. (Panel b) The same of the left panel for the log-likelihood $LL\left(\{I^{(\phi)}\},\{R_c\},\{\mu\},\overline{z},\sigma,V\right)$ with $\phi=\phi^{*}$.

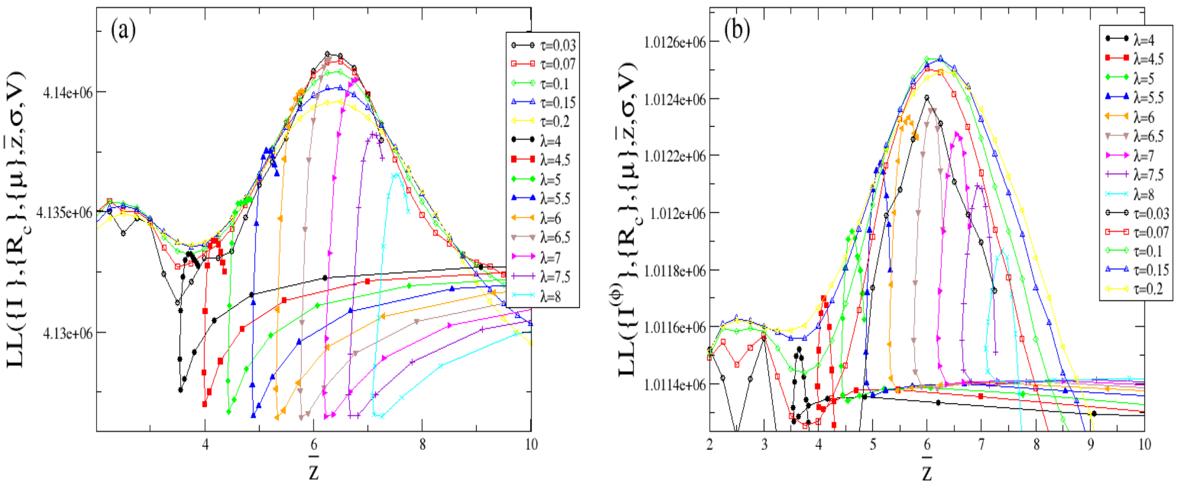

FIG. Supp.5: (Panel a) The log-likelihood $LL\left(\{I\},\{R_c\},\{\mu\},\overline{z},\sigma,V\right)$ for the optimal series $\{R_c\}$ and $\{\mu\}$, for a Weibull distributed $w(z)$ (filled symbols) and a Gamma distributed $w(z)$ (open symbols). Different curves correspond to different values of λ in the case of the Weibull distributed $w(z)$ and to different values of τ in the case of the Gamma distributed $w(z)$, as reported in the legend. (Panel b) The same of the left panel for the log-likelihood $LL\left(\{I^{(\phi)}\},\{R_c\},\{\mu\},\overline{z},\sigma,V\right)$ with $\phi=\phi^{*}$.

THE ROLE OF DIFFERENT LEARNING PERIODS

In this section we present the result of our procedure considering a learning period $m \in [1, m_{learning}]$. More precisely, we assume a Gamma distributed $w(z)$ and for different choices of the parameters $\overline{z}$ and σ we find the optimal series $\{R_c\}_{m=1,.,m_{learning}}$ and $\{\mu\}_{m=1,.,m_{learning}}$ which maximize the log-likelihood $LL(\{I\},\{R_c\},\{\mu\},\overline{z},\sigma,V)$ in the temporal window composed of $m_{learning}$ days starting form February 24 2020. Results for $LL(\{I\},\{R_c\},\{\mu\},\overline{z},\sigma,V)$ are plotted in Fig.Supp.6 for $m_{learning} = 10$ days and $m_{learning} = 30$ days. Fig.Supp.6 shows that, already after 10 days, $LL(\{I\},\{R_c\},\{\mu\},\overline{z},\sigma,V)$ presents a clear peak for $\overline{z} \in [5.8, 6]$ and $\sigma \in [0.8, 0.95]$, results in agreement with those found for a 1 year learning period.

The estimate of the optimal values of $\overline{z} \in [5.8, 6]$ and $\sigma \in [0.8, 0.95]$ obtained in the learning period can be used to estimate $R_c(m)$ and $\mu(m)$ in the following testing period $m > m_{learning}$. This information can be further used to generate synthetic sequences according to our numerical procedure (see Methods in the main text). Results of this procedure presented in Fig.Supp.7 show that, already for $m_{learning} = 10$ days, the simulated $\{I(m)\}$ is in very good agreement with the experimental one. In particular, the simulated $\{I(m)\}$ is able to capture also the daily fluctuations of the experimental incidence rate. This result indicates that these fluctuations do not have only a stochastic origin but also reflect genuine changes of the transmission rate adequately captured by $R_c(m)$. This demonstrates that our analysis is not affected by over-fitting. As further support, in Fig.Supp.7 we also show that implementing an erroneously estimated value of σ, during the learning period, leads to a much worst agreement during the testing period.

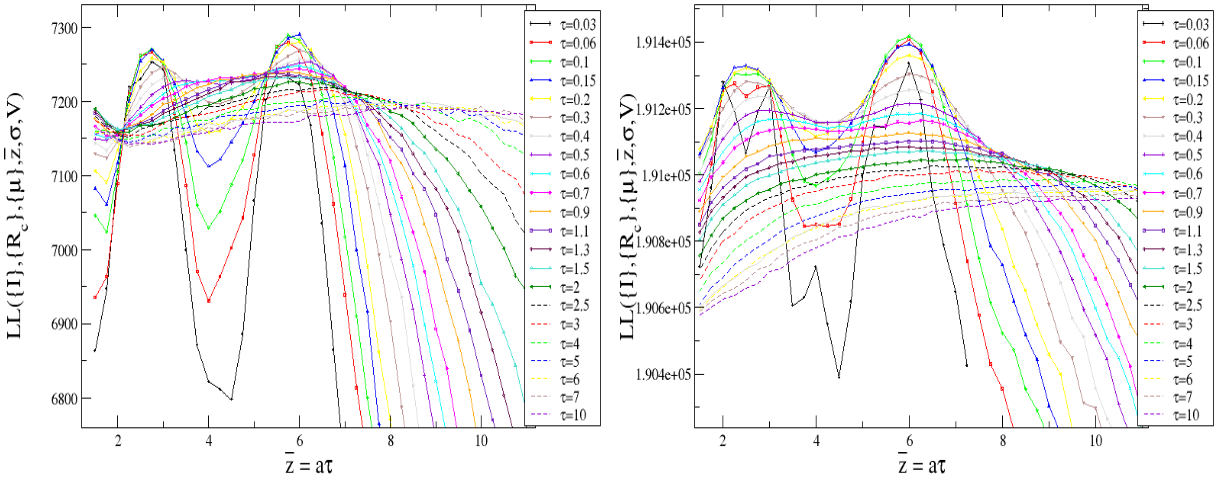

FIG. Supp.6: The log-likelihood $LL(\{I\},\{R_c\},\{\mu\},\overline{z},\sigma,V)$ for the optimal series $\{R_c\}$ and $\{\mu\}$ considering a learning period of 10 days (left panel) and 30 days (right panel). We implement a Gamma distributed $w(z)$.

THE DAILY INCIDENCE OF PATIENTS IN INTENSIVE CARE UNITS

In this section we apply our procedure considering for the incidence rate the daily number of individuals entering the intensive care units I^{ICU}. This information is available in Italy since December 2020 and we use one year of data up to December 2020. Since the daily value of I^{ICU} for a single region is usually small, we perform our analysis considering the I^{ICU} for the whole italian territory. The time evolution of $I^{ICU}(m)$ is plotted in the left panel of Fig.Supp.8. In the right panel of Fig.Supp.8 we perform the same analysis of Fig.3 of the manuscript considering the quantity $LL\left(\{I^{ICU}\},\{R_c\},\{\mu\},\overline{z},\sigma,V\right)$. Results confirm the best estimate for $\overline{z}z$ and σ obtained in the main text.

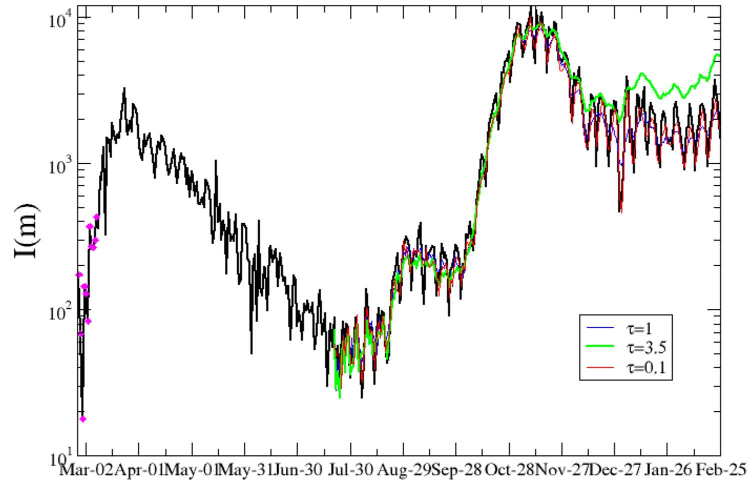

FIG. Supp.7: Black continuous lines represent the daily incidence of COVID-19 for Lombardy from 02/24/2020 to 02/24/2021. Magenta diamonds represent the result of numerical simulations implementing the best estimate for $\{R_c\}$ and $\{\mu\}$, for $\tau = 0.1$, $a = 6./\tau$, provided by the LL maximization procedure over the learning period of the first 10 days (up to 04/22/2020). The red color curve represents results of the numerical simulations implementing the best estimate for $\{R_c\}$ and $\{\mu\}$, over the testing period (m after 07/01/2020), using the values $\tau = 0.1$, $a = 6.0/\tau$ obtained during the learning period. The green (blue) curve represents results of the numerical simulations implementing the best estimate for $\{R_c\}$ and $\{\mu\}$, over the testing period (m after 07/01/2020), using the non optimal value $\tau = 3.5$ ($\tau = 1$) and $a = 6./\tau$.

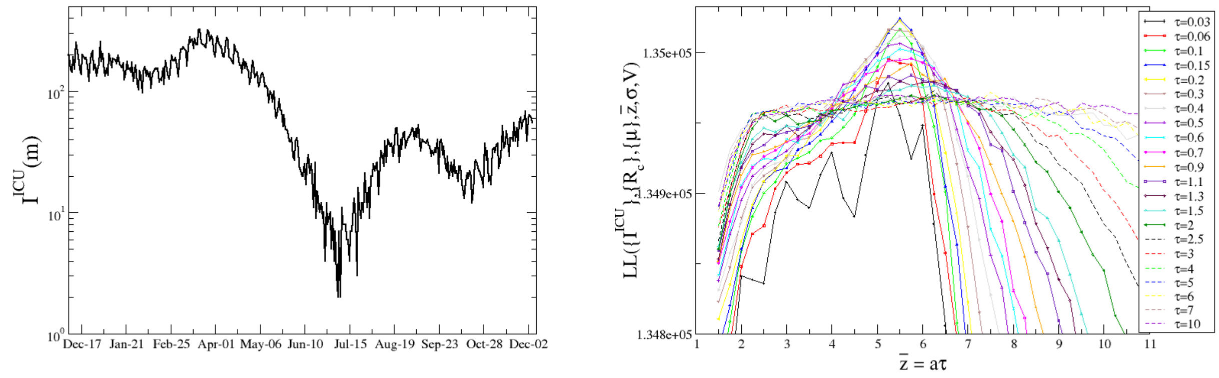

FIG. Supp.8: (Left panel) The daily number of patients entering intensive care units in Italy. (Right panel) The log-likelihood $LL\left(\{I^{ICU}\}, \{R_c\}, \{\mu\}, \overline{z}, \sigma, V\right)$ for the optimal series $\{R_c\}$ and $\{\mu\}$ considering the daily rate $I^{ICU}(m)$ plotted in the left panel. We implement a Gamma distributed $w(z)$.

THE OTHER ITALIAN REGIONS

In this section we present the same analysis performed in the main text for the region Lombardy for other five regions with the largest number of inhabitants, i.e. Lazio, Campania, Sicily, Veneto and Emilia-Romagna. The five regions are distributed over the italian territory, from North to South, and are therefore representative of the entire country. For each of the five regions we present a figure with four panels. In panel (a) we plot both the daily incidence rate $I(m)$ and the disentangled incidence rate $I^{(\phi)}(m)$ as function of m. In panel (b) we plot the best estimate of the reproduction number $R_c(m)$ and of $\mu(m)$ for the parameter of $w(z)$ leading to a maximum in $LL\left(\{I\},\{R_c\},\{\mu\},\overline{z},\sigma,V\right)$. In panel (c), we plot the $LL\left(\{I\},\{R_c\},\{\mu\},\overline{z},\sigma,V\right)$ for a Gamma distributed $w(z)$ ad function of $\overline{z}$ for different τ, or equivalently, different σ values. Finally in panel (d) we plot $LL\left(\{I^{(\phi)}\},\{R_c\},\{\mu\},\overline{z},\sigma,V\right)$ versus $\overline{z}$ for the disentangled incidence rate $\{I^{(\phi)}\}$ evaluated at $\phi=\phi^*$. All results are obtained implementing a Gamma distribution for $w(z)$ but we find similar results using a Weibull distribution. Very similar results are found for the other 15 Italian regions.

The different figures confirm the same result of the main text indicating a very peaked $w(z)$ with average value $\overline{z}$ presenting small fluctuations from region to region. A slightly different pattern is only obtained for $LL\left(\{I^{(\phi)},\{R_c\},\{\mu\},\overline{z},\sigma,V\right)$ for the Lazio and the Sicily regions, where the maximum at $\overline{z}\simeq 6$ days is still present but it is not the dominant one. Indeed, in Lazio the dominant maximum is at $\overline{z}\simeq 10$ days whereas it is at $\overline{z}\simeq 3$ days in Sicily. We do not have a clear explanation for this result but we could attribute the origin of the other maxima to some spurious periodicity introduced in the passage from $\{I\}$ to $\{I^{(\phi)}\}$.

A. Lazio

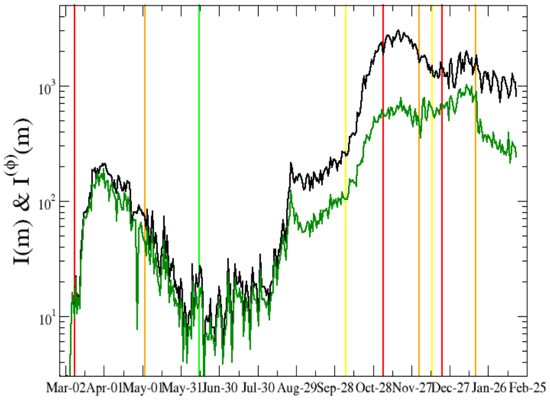

(a)

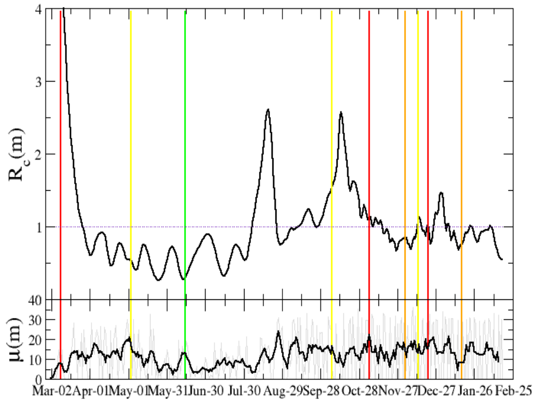

(b)

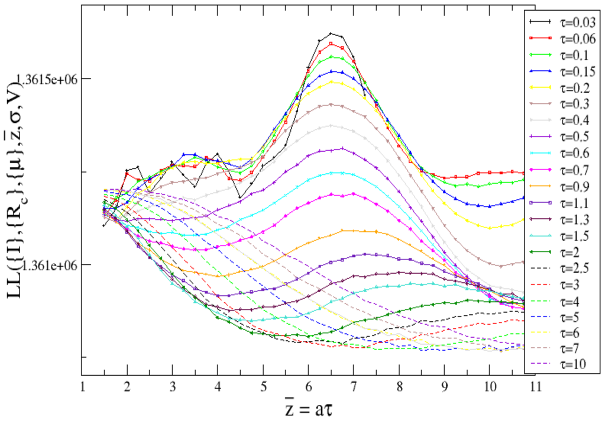

(c)

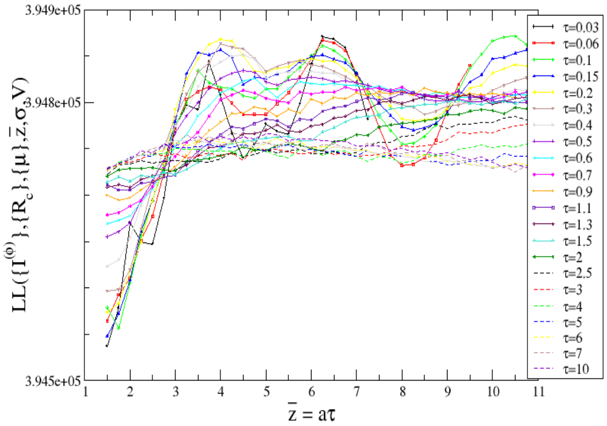

(d)

FIG. Supp.9: −(Panel a)− Black continuous lines represent the daily incidence $I(m)$ of COVID-19 in Lazio from 02/24/2020 up to 02/14/2021. Green continuous lines are used for the disentangled incidence rate $I^{(\phi)}(m)$ for $\phi = \phi^* = 1.1E - 3$. Color vertical lines indicate the starting time of different containment measures with a color code ranging from red, orange, yellow up to green as a rough indicator of the severity of these restrictions, decreasing from red to green, i.e strong restrictions are imposed in the temporal period after a red line whereas weak ones after a green one. −(Panel b)− (Upper Panel) The cohort reproduction number $R_c(m)$ of COVID-19 in Lazio from 02/24/2020 up to 02/14/2020 obtained by means of the LL maximization procedure for a Gamma distributed $w(z)$ at the peak value $\overline{z} = 6.5$ days and $\sigma = 0.80$ days. Color vertical lines indicate the starting time of different containment measures (see caption of panel (a)). (Lower Panel) The daily number of imported cases $\mu(m)$ estimated by the log-likelihood maximization procedure is plotted in thin grey whereas solid line is used for its weekly average. −(Panel c)− The log-likelihood $LL\left(\{I\}, \{R_c\}, \{\mu\}, \overline{z}, \sigma, V\right)$ obtained from the daily incidence of COVID-19 in Lazio, is plotted as a function of $\overline{z} = a\tau$. Different curves correspond to different values of τ (see legend) corresponding to different values of $\sigma = \sqrt{\tau \overline{z}}$. −(Panel d)−The log-likelihood $LL\left(\{I^{(\phi)}\}, \{R_c\}, \{\mu\}, \overline{z}, \sigma, V\right)$ obtained from the daily disentangled incidence of COVID-19 in Lazio, evaluated at $\phi = \phi^* = 1.1E - 3$, is plotted as a function of $\overline{z} = a\tau$. Different curves correspond to different values of τ (see legend) corresponding to different values of σ.

B. Campania

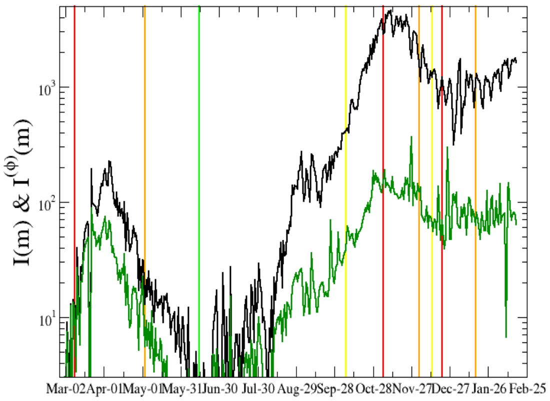

(a)

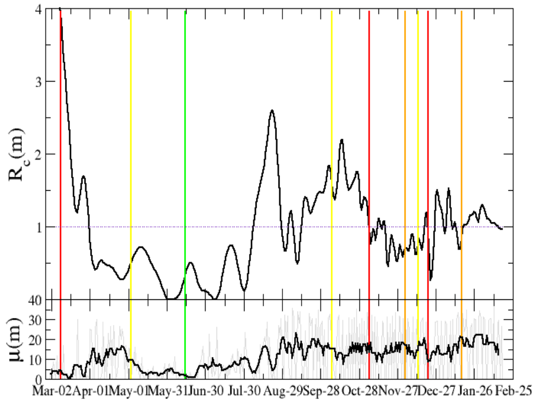

(b)

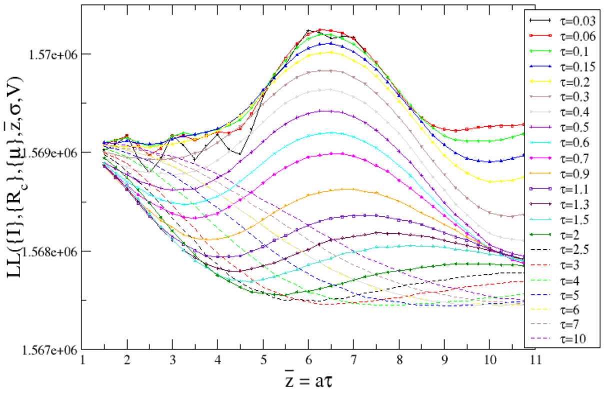

(c)

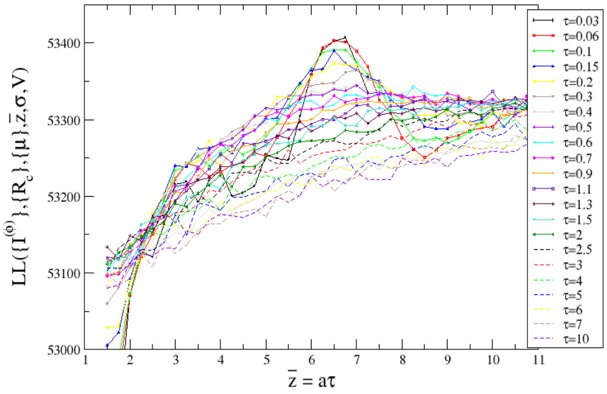

(d)

FIG. Supp.10: –(Panel a)– Black continuous lines represent the daily incidence $I(m)$ of COVID-19 in Campania from 02/24/2020 up to 02/14/2021. Green continuous lines are used for the disentangled incidence rate $I^{(\phi)}(m)$ for $\phi = \phi^* = 1E-5$. Color vertical lines indicate the starting time of different containment measures with a color code ranging from red, orange, yellow up to green as a rough indicator of the severity of these restrictions, decreasing from red to green, i.e strong restrictions are imposed in the temporal period after a red line whereas weak ones after a green one. –(Panel b)– (Upper Panel) The cohort reproduction number $R_c(m)$ of COVID-19 in Campania from 02/24/2020 up to 02/14/2020 obtained by means of the LL maximization procedure for a Gamma distributed $w(z)$ at the peak value $\overline{z} = 6$ days and $\sigma = 0.77$ days. Color vertical lines indicate the starting time of different containment measures (see caption of panel (a)). (Lower Panel) The daily number of imported cases $\mu(m)$ estimated by the log-likelihood maximization procedure is plotted in thin grey whereas solid line is used for its weekly average. –(Panel c)– The log-likelihood $LL\left(\{I\},\{R_c\},\{\mu\},\overline{z},\sigma,V\right)$ obtained from the daily incidence of COVID-19 in Campania, is plotted as a function of $\overline{z} = a\tau$. Different curves correspond to different values of τ (see legend) corresponding to different values of $\sigma = \sqrt{\tau\overline{z}}$. –(Panel d)–The log-likelihood $LL\left(\{I^{(\phi)}\},\{R_c\},\{\mu\},\overline{z},\sigma,V\right)$ obtained from the daily disentangled incidence of COVID-19 in Campania, evaluated at $\phi = \phi^* = 1.1E-3$, is plotted as a function of $\overline{z} = a\tau$. Different curves correspond to different values of τ (see legend) corresponding to different values of σ.

C. Sicily

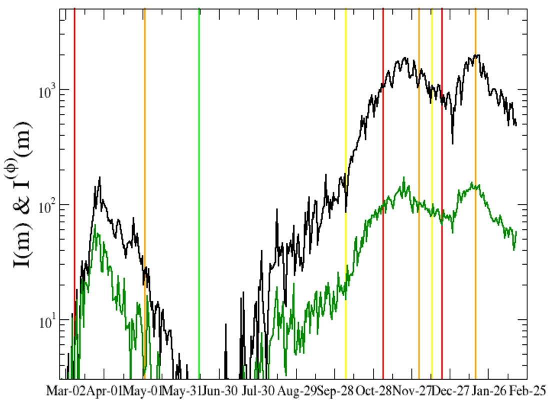

(a)

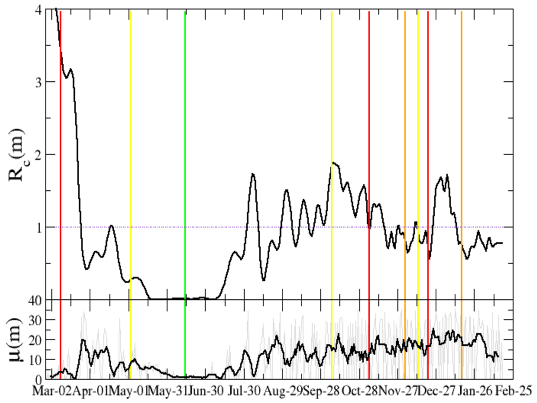

(b)

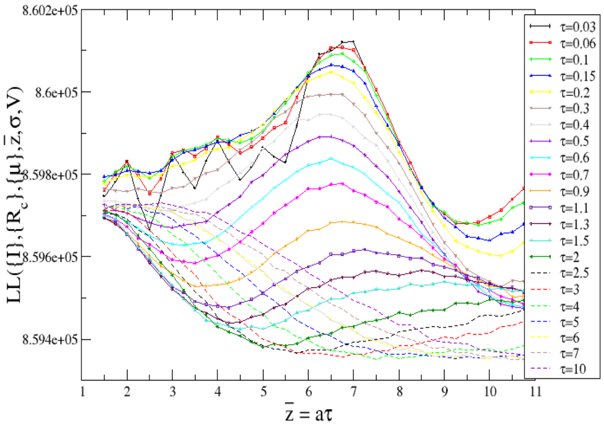

(c)

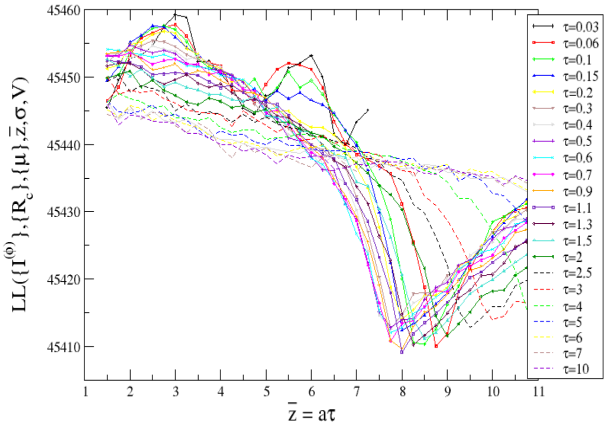

(d)

FIG. Supp.11: −(Panel a)− Black continuous lines represent the daily incidence $I(m)$ of COVID-19 in Sicily from 02/24/2020 up to 02/14/2021. Green continuous lines are used for the disentangled incidence rate $I^{(\phi)}(m)$ for $\phi = \phi^* = 1.1E-4$. Color vertical lines indicate the starting time of different containment measures with a color code ranging from red, orange, yellow up to green as a rough indicator of the severity of these restrictions, decreasing from red to green, i.e strong restrictions are imposed in the temporal period after a red line whereas weak ones after a green one. −(Panel b)− (Upper Panel) The cohort reproduction number $R_c(m)$ of COVID-19 in Sicily from 02/24/2020 up to 02/14/2020 obtained by means of the LL maximization procedure for a Gamma distributed $w(z)$ at the peak value $\overline{z} = 6.75$ days and $\sigma = 0.82$ days. Color vertical lines indicate the starting time of different containment measures (see caption of panel (a)). (Lower Panel) The daily number of imported cases $\mu(m)$ estimated by the log-likelihood maximization procedure is plotted in thin grey whereas solid line is used for its weekly average. −(Panel c)− The log-likelihood $LL\left(\{I\},\{R_c\},\{\mu\},\overline{z},\sigma,V\right)$ obtained from the daily incidence of COVID-19 in Sicily, is plotted as a function of $\overline{z} = a\tau$. Different curves correspond to different values of τ (see legend) corresponding to different values of $\sigma = \sqrt{\tau\overline{z}}$. −(Panel d)−The log-likelihood $LL\left(\{I^{(\phi)}\},\{R_c\},\{\mu\},\overline{z},\sigma,V\right)$ obtained from the daily disentangled incidence of COVID-19 in Sicily, evaluated at $\phi = \phi^* = 1.1E-3$, is plotted as a function of $\overline{z} = a\tau$. Different curves correspond to different values of τ (see legend) corresponding to different values of σ.

D. Veneto

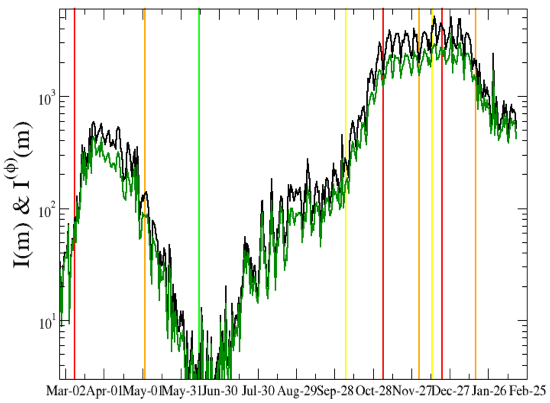

(a)

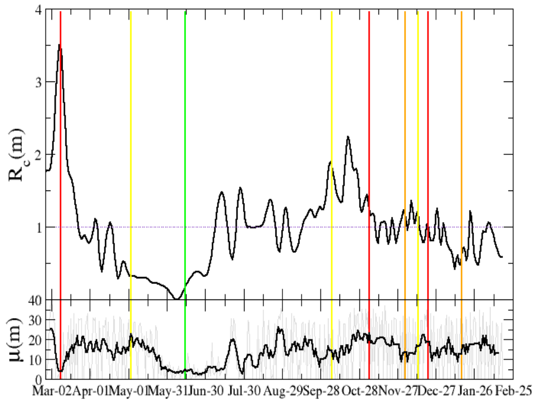

(b)

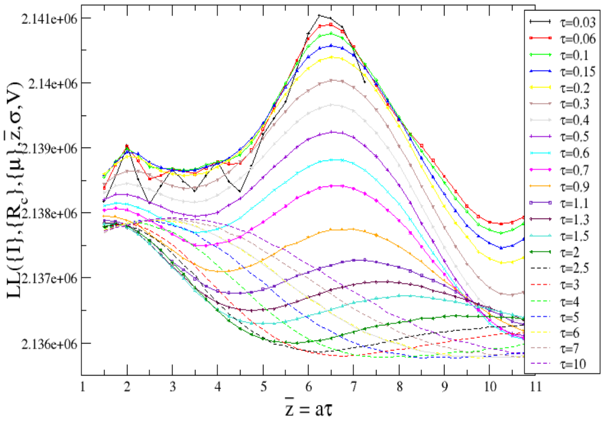

(c)

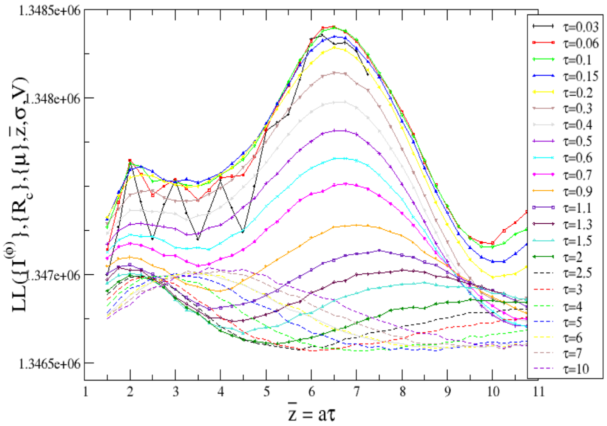

(d)

FIG. Supp.12: −(Panel a)− Black continuous lines represent the daily incidence $I(m)$ of COVID-19 in Veneto from 02/24/2020 up to 02/14/2021. Green continuous lines are used for the disentangled incidence rate $I^{(\phi)}(m)$ for $\phi = \phi^* = 2E-3$. Color vertical lines indicate the starting time of different containment measures with a color code ranging from red, orange, yellow up to green as a rough indicator of the severity of these restrictions, decreasing from red to green, i.e strong restrictions are imposed in the temporal period after a red line whereas weak ones after a green one. −(Panel b)− (Upper Panel) The cohort reproduction number $R_c(m)$ of COVID-19 in Veneto from 02/24/2020 up to 02/14/2020 obtained by means of the LL maximization procedure for a Gamma distributed $w(z)$ at the peak value $\overline{z} = 6.25$ days and $\sigma = 0.79$ days. Color vertical lines indicate the starting time of different containment measures (see caption of panel (a)). (Lower Panel) The daily number of imported cases $\mu(m)$ estimated by the log-likelihood maximization procedure is plotted in thin grey whereas solid line is used for its weekly average. −(Panel c)− The log-likelihood $LL\left(\{I\},\{R_c\},\{\mu\},\overline{z},\sigma,V\right)$ obtained from the daily incidence of COVID-19 in Veneto, is plotted as a function of $\overline{z} = a\tau$. Different curves correspond to different values of τ (see legend) corresponding to different values of $\sigma = \sqrt{\tau\overline{z}}$. −(Panel d)−The log-likelihood $LL\left(\{I^{(\phi)}\},\{R_c\},\{\mu\},\overline{z},\sigma,V\right)$ obtained from the daily disentangled incidence of COVID-19 in Veneto, evaluated at $\phi = \phi^* = 1.1E-3$, is plotted as a function of $\overline{z} = a\tau$. Different curves correspond to different values of τ (see legend) corresponding to different values of σ.

E. Emilia-Romagna

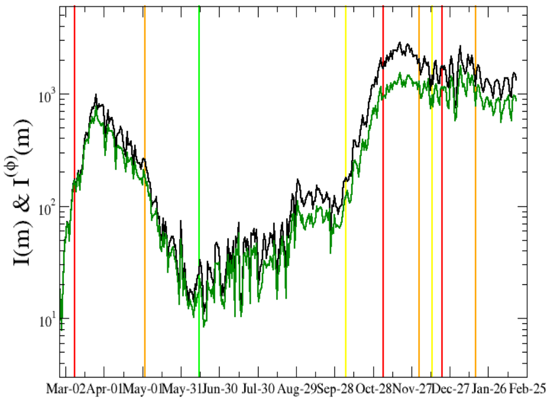

(a)

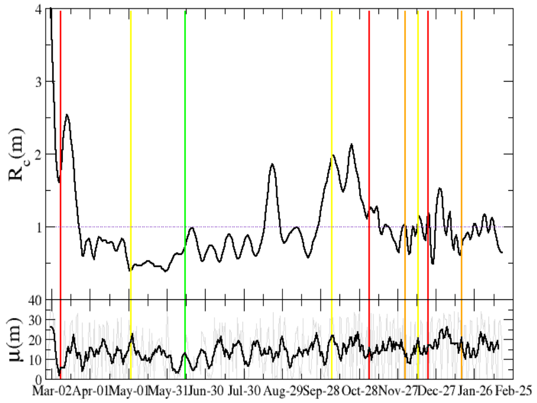

(b)

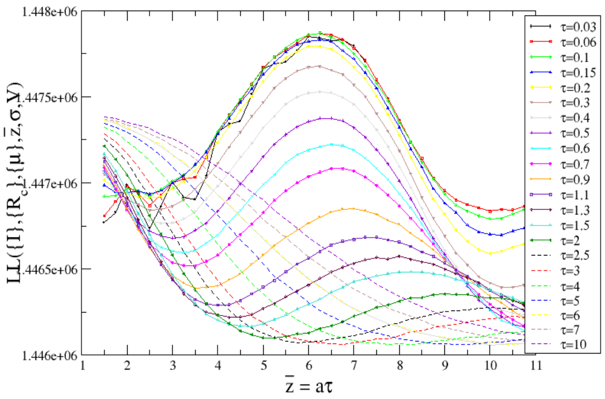

(c)

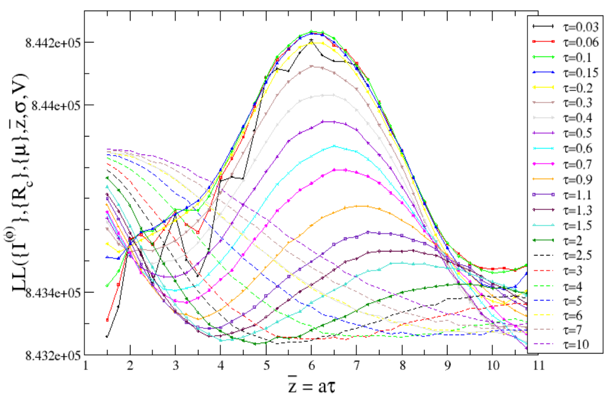

(d)

FIG. Supp.13: –(Panel a)– Black continuous lines represent the daily incidence $I(m)$ of COVID-19 in Emilia-Romagna from 02/24/2020 up to 02/14/2021. Green continuous lines are used for the disentangled incidence rate $I^{(\phi)}(m)$ for $\phi = \phi^* = 2E-3$. Color vertical lines indicate the starting time of different containment measures with a color code ranging from red, orange, yellow up to green as a rough indicator of the severity of these restrictions, decreasing from red to green, i.e strong restrictions are imposed in the temporal period after a red line whereas weak ones after a green one. –(Panel b)– (Upper Panel) The cohort reproduction number $R_c(m)$ of COVID-19 in Emilia-Romagna from 02/24/2020 up to 02/14/2020 obtained by means of the LL maximization procedure for a Gamma distributed $w(z)$ at the peak value $\overline{z} = 6.5$ days and $\sigma = 0.81$ days. Color vertical lines indicate the starting time of different containment measures (see caption of panel (a)). (Lower Panel) The daily number of imported cases $\mu(m)$ estimated by the log-likelihood maximization procedure is plotted in thin grey whereas solid line is used for its weekly average. –(Panel c)– The log-likelihood $LL(\{I\}, \{R_c\}, \{\mu\}, \overline{z}, \sigma, V)$ obtained from the daily incidence of COVID-19 in Emilia-Romagna, is plotted as a function of $\overline{z} = a\tau$. Different curves correspond to different values of τ (see legend) corresponding to different values of $\sigma = \sqrt{\tau \overline{z}}$. –(Panel d)–The log-likelihood $LL\left(\{I^{(\phi)}\}, \{R_c\}, \{\mu\}, \overline{z}, \sigma, V\right)$ obtained from the daily disentangled incidence of COVID-19 in Emilia-Romagna, evaluated at $\phi = \phi^* = 1.1E-3$, is plotted as a function of $\overline{z} = a\tau$. Different curves correspond to different values of τ (see legend) corresponding to different values of σ.
